# Bat organoids reveal antiviral responses at epithelial surfaces

**DOI:** 10.1038/s41590-025-02155-1

**Published:** 2025-05-21

**Authors:** Max J. Kellner, Vanessa M. Monteil, Patrick Zelger, Gang Pei, Jie Jiao, Masahiro Onji, Komal Nayak, Matthias Zilbauer, Anne Balkema-Buschmann, Anca Dorhoi, Ali Mirazimi, Josef M. Penninger

**Affiliations:** 1 Institute of Molecular Biotechnology of the Austrian Academy of Sciences (IMBA), Vienna BioCenter (VBC), Vienna, Austria; 2 Vienna BioCenter PhD Program, Doctoral School of the University of Vienna and Medical University of Vienna, Vienna, Austria; 3 Department of Laboratory Medicine, Medical University of Vienna, Vienna, Austria; 4 Division of Clinical Microbiology, Department of Laboratory Medicine, Karolinska Institute and Karolinska University Hospital, Stockholm, Sweden; 5 Institute of Immunology, Friedrich-Loeffler-Institut, Greifswald-Insel Riems, Germany; 6 Department of Medical Genetics, Life Sciences Institute, University of British Columbia, Vancouver, British Columbia Canada; 7 Wellcome – MRC Cambridge Stem Cell Institute, University of Cambridge, Cambridge, UK; 8 Department of Paediatrics, University of Cambridge, Cambridge, UK; 9 Institute of Novel and Emerging Infectious Diseases, Friedrich-Loeffler-Institut, Greifswald-Insel Riems, Germany; 10 Faculty of Mathematics and Natural Sciences, University of Greifswald, Greifswald, Germany; 11 National Veterinary Institute, Uppsala, Sweden; 12 Public Health Agency of Sweden, Solna, Sweden; 13 Helmholtz Centre for Infection Research, Braunschweig, Germany

**Keywords:** Mucosal immunology, Viral infection

## Abstract

Bats can host viruses of pandemic concern without developing disease. The mechanisms underlying their exceptional resilience to viral infections are largely unresolved, necessitating the development of physiologically relevant and genetically tractable research models. Here, we developed respiratory and intestinal organoids that recapitulated the cellular diversity of the in vivo epithelium present in *Rousettus aegyptiacus*, the natural reservoir for the highly pathogenic Marburg virus (MARV). In contrast to human counterparts, bat organoids and mucosal tissue exhibited elevated constitutive expression of innate immune effectors, including type I interferon-ε (IFNε) and IFN-stimulated genes (ISGs). Upon infection with diverse zoonotic viruses, including MARV, bat organoids strongly induced type I and III IFN responses, which conferred robust antiviral protection. Type III IFNλ3 additionally displayed virus-independent self-amplification, acting as an ISG to enhance antiviral immunity. Our organoid platform reveals key features of bat epithelial antiviral immunity that may inform therapeutic strategies for viral disease resilience.

## Abstract (web summary)

Kellner et al. develop respiratory and intestinal organoids from *Rousettus aegyptiacus* to show elevated basal expression of interferon-ε (IFNε) and IFN-stimulated genes, along with robust, self-amplifying type III IFN responses that drive antiviral defense against zoonotic RNA viruses.

## Main

Bats possess a unique ability to host and tolerate pathogens that are highly virulent to humans and nonhuman primates^1^. Insights from comparative genomic studies in bats have suggested a genetic basis for their exceptional immunity, supported by positive selection or loss of genes that could enhance innate immune responses and limit overt inflammation^2–5^. However, functional genetic studies in bats remain a challenging task owing to their unique lifestyle, protected status and the limited molecular tools developed and optimized for these non-model organisms^6^. Pioneering research on bat antiviral immunity has largely focused on peripheral immune responses in infected bats or immortalized cell lines, which have provided crucial insights into their immune defense mechanisms^7–9^. However, mucosal surfaces, which serve as primary sites for viral entry and form the first line of antiviral defense against both local and systemic infections, have not been thoroughly studied in bats^10^.

In this study, we developed a sustainable organoid platform that accurately models the respiratory and small intestinal (SI) epithelia of *Rousettus aegyptiacus* (Egyptian fruit bat), a natural reservoir for several human pathogens, including the highly lethal Marburg virus (MARV)^7,11–13^. Through single-cell RNA sequencing (scRNA-seq), viral infection and genetic perturbation experiments, we uncovered a heightened constitutive expression of innate immune effector genes and enhanced IFN responses to zoonotic viruses in *R. aegyptiacus* epithelial organoids compared to human counterparts. We further delineated the role of type I and III IFNs in providing robust and long-lasting antiviral protection. These findings establish a valuable resource for studying antiviral immunity at bat epithelial surfaces and reveal species-specific immune adaptations that may underlie bat resilience to emerging zoonotic viruses.

## Results

### *R. aegyptiacus* airway organoids contain diverse cell types

**Fig. 1:**
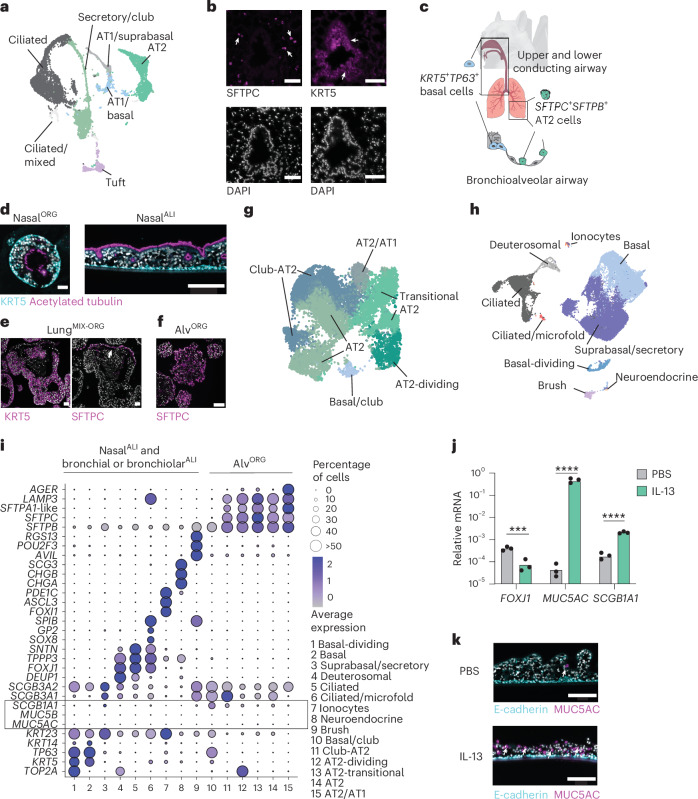
*R. aegyptiacus* nasal, bronchial and alveolar airway organoids contain diverse cell types. **a**, Uniform manifold approximation and projection (UMAP) of EC types of lung and tracheal tissue, identified using scRNA-seq from a captive-bred *R. aegyptiacus*. Clusters labeled with ‘/’ indicate mixed identities: AT1/basal (AT1 lung or basal cells from the lung or trachea); AT1/suprabasal (AT1 lung or suprabasal trachea); secretory/club (goblet or club cells); ciliated/mixed (cells with ambiguous identity expressing a ciliated marker). **b**, Representative KRT5 or SFTPC immunofluorescence staining (top) and 4′,6-diamidino-2-phenylindole (DAPI) nucleus counterstaining (bottom) in *R. aegyptiacus* lung sections (*n* = 3). Arrowheads indicate KRT5^+^ basal or SFTPC^+^ AT2 cells. Scale bars, 50 µm. **c**, Schematic of the main adult respiratory epithelial stem cell types: *KRT5*^+^*TP63*^+^ basal cells in the upper and lower conducting airways and *SFTPC*^+^*SFTPB*^*+*^ AT2 cells in the alveolar epithelium. **d**, Representative KRT5 and acetylated tubulin immunofluorescence staining in differentiated nasal^ORG^ (left) and nasal^ALI^ (right) cultures derived from *R. aegyptiacus*. Scale bars, 50 µm (left) or 150 µm (right). **e**, Representative KRT5 (left) or SFTPC (right) immunofluorescence staining in lung^MIX-ORG^ derived from *R. aegyptiacus* and grown in complete alveolar medium (Methods). The arrowhead highlights SFTPC^+^ cells. Scale bars, 50 µm. **f**, Representative SFTPC immunofluorescence staining in alv^ORG^ derived from *R. aegyptiacus*. Scale bar, 100 µm. **g**, UMAP of the *R. aegyptiacus*-derived alv^ORG^ scRNA-seq dataset showing the cell-type clusters. **h**, UMAP of the integrated *R. aegyptiacus*-derived nasal^ALI^ and bronchial^ALI^ scRNA-seq dataset showing the cell-type clusters. **i**, Seurat DotPlot showing the average expression of the markers for each cell cluster in a merged dataset of *R. aegyptiacus*-derived nasal^ALI^ + bronchial^ALI^ and alv^ORG^. The dot size represents the percentage of a cell type expressing a given marker. Color intensity represents the average expression value. Dot size was set to a maximum percentage of 50%. (Genes expressed by more than 50% of cells have the same dot size.) The box highlights the low-to-absent expression of the club and goblet cell markers *MUC5AC* and *MUC5B*. **j**, RT–qPCR analysis of *FOXJ1*, *MUC5AC* or *SCGB1A1* expression (*n* = 3 each; normalized to *EEF1A1*, 2^−^^Δ*C*t^) in *R. aegyptiacus*-derived nasal^ALI^ treated with 10 ng ml^−1^ recombinant human IL-13 or PBS from days 10 to 25 of the ALI culture. **k**, Representative immunofluorescence staining of MUC5AC and E-cadherin in sections from *R. aegyptiacus*-derived nasal^ALI^ treated with IL-13 or PBS as in **i**. Scale bars, 150 µm. Representative images in **d**–**f**,**k** were derived from *n* = 3. DAPI was used as a nuclear counterstain in immunofluorescence imaging.

**Extended Data Fig. 1:**
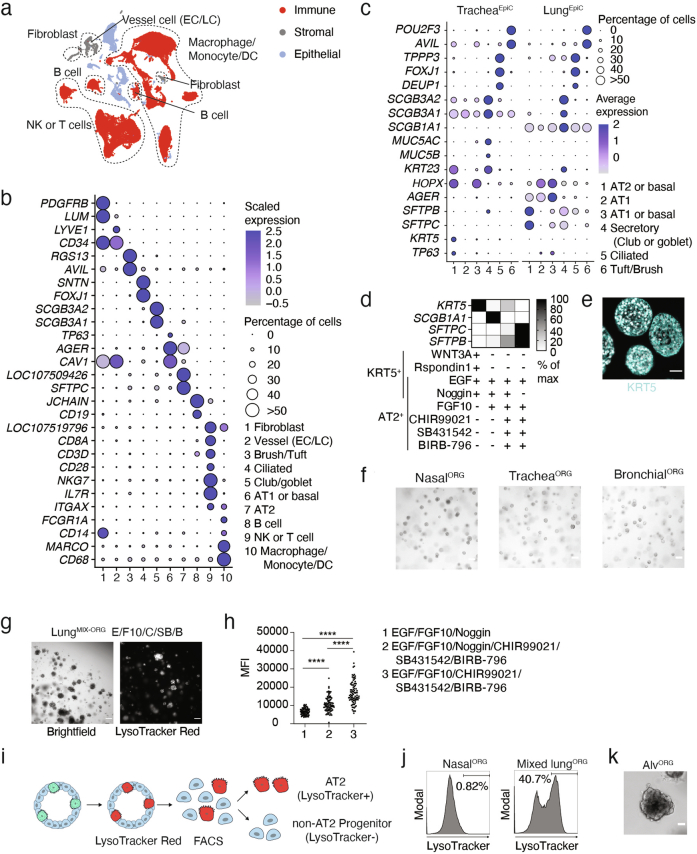
Establishment and characterization of *R. aegyptiacus* airway organoids. **a**, UMAP of *R. aegyptiacus* airway cell types resolved by scRNA-seq of lung and trachea tissue from a captive-bred bat. Immune, stromal, and epithelial cell lineages are labeled and color-coded. EC, Endothelial cells, LC, Lymphatic cells, DC, Dendritic cells. **b**, Seurat *DotPlot* showing scaled average expression of marker genes for cell clusters in **(a)**, grouped by major tissue-resident lineages. Dot sizes were set to a maximum percentage of 50% (genes expressed by more than 50% of cells have the same dot size). Color intensity represents expression level. **c**, Seurat *DotPlot* showing marker expression across epithelial cell types. Dot sizes were set to a maximum percentage of 50%. **d**, Heatmap showing RT-qPCR analysis of *KRT5*, *SCGB1A1*, *SFTPC*, or *SFTPB* in mixed lung progenitor organoids (Lung^MIX-ORG^) cultured with indicated factors. Expression normalized to *EEF1A1* and shown as % of maximum. Each value represents the average relative expression (n = 3). **e**, Representative immunofluorescence of KRT5 in bat nasal^ORG^ grown in basal cell organoid medium (EGF, FGF10, Rspondin1, Noggin) (n = 3). Composite image with DAPI nuclei counter stain. Scale, 50 µm. **f**, Representative brightfield images of nasal^ORG^, trachea^ORG^, or bronchial^ORG^ at day four following single-cell passage (n = 3). Scale, 100 µm. **g**, Representative brightfield (left) and fluorescence image (right) of bat lung^MIX-ORG^ grown in complete alveolar medium (-Noggin) stained with LysoTracker-Red for 2 hours (n = 3 each). Scale, 100 µm. **h**, Mean Fluorescence Intensity (MFI) of LysoTracker Red staining in mixed lung^MIX-ORG^ (n = 3) cultured with indicated factors. Dots, image intensity per single organoid analyzed in *Fiji*. Two-sided unpaired Mann-Whitney tests were performed to compare MFI distribution of lung^MIX-ORG^ cultured in different media (**** P < 0.0001). **i**, Schematic showing alv^ORG^ derivation from LysoTracker Red^+^ AT2 FACS-sorted cells from lung^MIX-ORG^. **j**, FACS plots showing LysoTracker Red intensity in single gated cells from nasal^ORG^ or lung^MIX-ORG^ cultured in complete alveolar medium. Percentages of LysoTracker^+^ cells are shown. **k**, Representative brightfield image of bat alv^ORG^ established from FACS-sorted AT2 cells. Scale, 100 µm.

The mammalian respiratory epithelium has a central role in orchestrating immune responses to viral infections^14^. We aimed to generate bat adult stem cell-derived epithelial organoids from the upper and lower respiratory tract of *R. aegyptiacus* as the model species. To establish a tissue reference dataset, we performed integrative scRNA-seq on whole trachea and lung tissue fragments from a captive-bred *R. aegyptiacus* and identified distinct clusters of immune, stromal and epithelial cell (EC) lineages (Extended Data Fig. 1a,b and Supplementary Table 1). Among EC clusters, we identified two progenitor stem cell types, namely *KRT5*^+^*TP63*^+^ basal cells, predominantly found in the trachea, and *SFTPC*^+^*SFTPB*^+^ alveolar type 2 (AT2) cells, which were exclusively present in the lung (Fig. 1a and Extended Data Fig. 1c). Differentiated epithelial cell lineages included *MUC5AC*^+^*MUC5B*^+^ secretory goblet and club cells (*SCGB1A1*, *SCGB3A1* and *SCGB3A2*), ciliated cells (*FOXJ1*, *SNTN* and *TPPP3*), brush cells (*POU2F3*, *AVIL* and *RGS13*) and alveolar type I (AT1) cells (*AGER*, *HOPX* and *CAV1*) (Fig. 1a and Extended Data Fig. 1c). Immunofluorescence staining of bat lung showed that *KRT5*^+^ basal cells localized to conducting bronchial and bronchiolar airway structures, while *SFTPC*⁺ AT2 cells were distributed throughout the lung parenchyma (Fig. 1b). The Egyptian fruit bat respiratory airway epithelium thus contains at least two different progenitor cell types, *KRT5*^+^*TP63*^+^ basal cells of the upper and lower conducting airway and *SFTPC*^+^*SFTPB*^+^ AT2 cells of the lung (Fig. 1c).

Because a major bottleneck was access to fresh bat tissue, we established a protocol for effective cryopreservation of primary bat tissue, enabling shipping and subsequent use as the starting material for organoid derivation (Methods). Through empirical testing of growth factors known to support the proliferation of adult airway stem cells in vitro^15,16^, we identified serum-free medium compositions that promoted long-term expansion of basal cell-derived and alveolar cell-derived organoids for at least 6 months (Extended Data Fig. 1d). KRT5^+^ basal cell organoids derived from nasal, tracheal or bronchial and bronchiolar lung tissue of *R. aegyptiacus* (designated nasal^ORG^, tracheal^ORG^ and bronchial^ORG^) exhibited compact morphology when grown in expansion medium (Extended Data Fig. 1e) and formed a lumen with inward-facing, beating cilia on switching to differentiation medium (Fig. 1d). To obtain well-differentiated organotypic cultures, we also differentiated nasal^ORG^-derived or bronchial^ORG^-derived cells at the air–liquid interface (ALI) (nasal^ALI^ or bronchial^ALI^) (Fig. 1d). Bat lung alveolar organoids (alv^ORG^) were established from sorted alveolar AT2 cells of early-passage mixed progenitor lung organoids (lung^MIX-ORG^), containing bronchial and bronchiolar basal and AT2 cells; Fig. 1e) using LysoTracker Red, a cell-permeable dye that labels lamellar bodies in AT2 cells^17^ (Extended Data Fig. 1g–j). Bat alv^ORG^ exhibited a saccular morphology consisting of SFTPC^+^ AT2 cells (Fig. 1f and Extended Data Fig. 1k) and could be serially passaged for at least 6 months. Notably, we successfully established and characterized organoids from independently frozen tissue samples from three different *R. aegyptiacus* bats with similar results.

**Extended Data Fig. 2:**
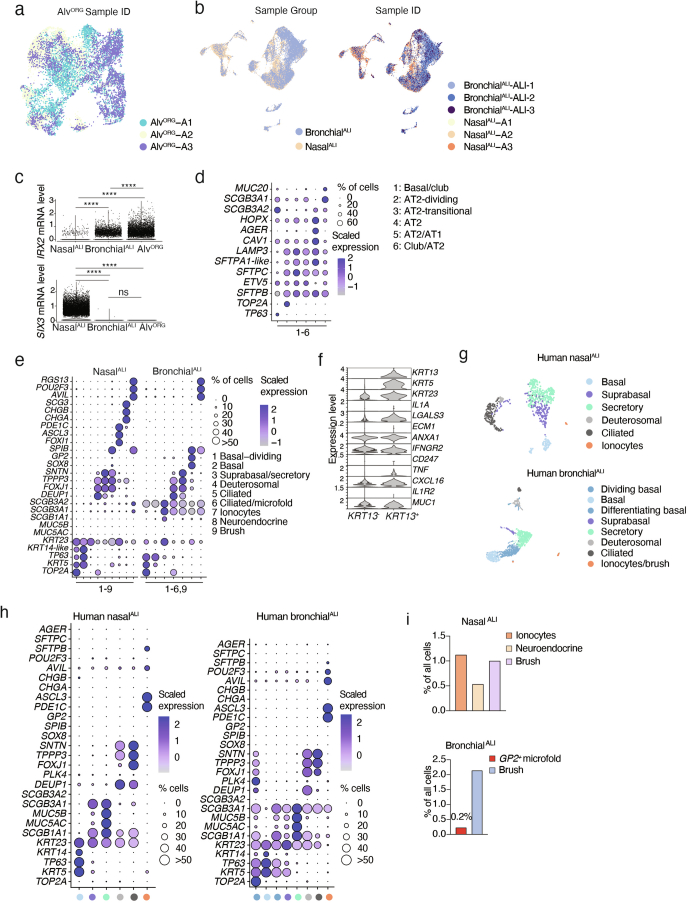
Single-cell RNA sequencing of bat and human airway organoids. **a**, UMAP of alv^ORG^ cell types resolved by scRNA-seq, colored by bat donor (n = 3). **b**, UMAP of bat nasal^ALI^ and bronchial^ALI^ cell types by sample group (left, nasal^ALI^ or bronchial^ALI^) or donor (right, n = 3). **c**, Seurat *VlnPlots* showing *IRX2* (top) and *SIX3* (bottom) expression in bat nasal^ALI^, bronchial^ALI^, or alv^ORG^. Wilcoxon Rank Sum Test used (**** *P* < 0.0001). **d**, Seurat *DotPlot* showing marker expression in alv^ORG^ clusters. Dot sizes were set to a maximum percentage of 50% (genes expressed by more than 50% of cells have the same dot size). Dot color intensity represents scaled expression level. **e**, Same as **(d)** for bat nasal^ALI^ and bronchial^ALI^. **f**, Seurat *VlnPlot* showing *KNIIFE-cell* markers in *KRT13*^+^ vs. *KRT13*^−^ cells in the Nasal^ALI^ scRNA-seq dataset. **g**, UMAP of human nasal^ALI^ (top) or bronchial^ALI^ (bottom) airway cell types resolved by scRNA-seq. Cell types are labeled and color-coded. **h**, Seurat *DotPlot* marker gene expression human nasal^ALI^ (left) or bronchial^ALI^ (right). Dot sizes were set to a maximum percentage of 50%. Dot color intensity represents scaled expression level. **i**, Bar plots showing the percentages of rare cell types relative to all cells in the bat nasal^ALI^ (top) or bronchial^ALI^ (bottom) scRNA-seq dataset.

To evaluate the cellular diversity of organoids, we subjected bat alv^ORG^, or bat nasal^ALI^ and bronchial^ALI^, to scRNA-seq. Integrative scRNA-seq analyses of bat alv^ORG^ or bat nasal^ALI^ and bronchial^ALI^ showed that cells clustered according to cell type rather than culture model or individual bat (Fig. 1g,h, Extended Data Fig. 2a,b and Supplementary Table 2). scRNA-seq further revealed that bat alv^ORG^, nasal^ALI^ and bronchial^ALI^ retained the expression patterns of prototypical regional homeobox transcription factors, such as a gradient of increasing expression of *IRX2* (ref. ^18^) from nasal^ALI^ to bronchial^ALI^ to alv^ORG^, or the exclusive expression of *SIX3* (ref. ^19^) in bat nasal^ALI^ (Extended Data Fig. 2c), highlighting the preservation of positional memory after extended in vitro culture. In alv^ORG^, we observed an expected enrichment of AT2 cell lineages (*SFTPC*, *SFTPB*, *SFTPA1-*like (*LOC107509426*)), annotated as AT2, dividing AT2 (coexpressing *TOP2A*) and transitional AT2 (*AGER*^lo^, *HOPX*^lo^), over *TP63*^+^ basal cells (Fig. 1g,i). Alv^ORG^ also consisted of *SFTPC*^+^*SFTPB*^+^ AT2 cells that additionally expressed secretory or club cell genes (*SCGB3A2*, *MUC20*; Club/AT2) or markers of AT1 cells (*AGER*^hi^*HOPX*^hi^,*CAV1*^hi^; AT2/AT1) (Fig. 1g,i and Extended Data Fig. 2d). Among cell-type clusters from bat nasal^ALI^ or bronchial^ALI^, we identified basal cells characterized by the expression of *TP63* and *KRT5*, most of which also coexpresssed *KRT14* (encoded by *LOC107513879*) in nasal^ALI^ or *SCGB3A2* in bronchial^ALI^ (Fig. 1h,i and Extended Data Fig. 2e). Further classification enabled us to distinguish suprabasal and secretory cells in nasal^ALI^ (*KRT5*^lo^, *TP63*^lo^, *KRT23*, *PIGR)* or bronchial^ALI^ (*KRT5*^lo^, *TP63*^lo^, *KRT23*, *SCGB3A1*, *SCGB3A2*), deuterosomal (*TOP2A*, *DEUP1*, *FOXJ1*) and ciliated cells (*FOXJ1*, *TPPP3*, *SNTN*) (Fig. 1h,i and Extended Data Fig. 2e). We also identified rare brush and tuft cells (*AVIL*, *RGS13*, *POU2F3*) in bat nasal^ALI^ or bronchial^ALI^ (Fig. 1h,i and Extended Data Fig. 2e). These cells have a crucial role in innate immunity by detecting pathogens and producing cytokines such as interleukin-25, as well as lipid inflammatory mediators like cysteinyl leukotrienes^20^. Additionally, we uncovered cells expressing markers of ionocytes (*ASLC3*, *FOXI1*, *PDE1C*) and neuroendocrine cells (*CHGA*, *CHGB*, *SCG3*) in nasal^ALI^ (Fig. 1h,i and Extended Data Fig. 2e). *KRT13*^+^*IL1A*^+^*KRT5*^−^ nasal immune-interacting floor-epithelial-like cells, which were recently described in mice^21^ but are absent in human nasal respiratory tissue^22^, were not identified in the bat nasal^ALI^ scRNA-seq dataset (Extended Data Fig. 2f).

We observed little to no secretory *MUC5B*^+^*MUC5AC*^+^ goblet cells in bat nasal^ALI^ or bronchial^ALI^ (Fig. 1i and Extended Data Fig. 2e), in contrast to human nasal^ALI^ or bronchial^ALI^, prepared in parallel using identical culture conditions (Extended Data Fig. 2g,h and Supplementary Table 3). To test whether *R. aegyptiacus* basal cells had the intrinsic capacity to differentiate into goblet cells in vitro, we stimulated bat nasal^ALI^ cultures with the type 2 immunity-associated cytokine interleukin-13 (IL-13), which in humans drives goblet cell metaplasia resulting in an altered ciliated:secretory cell ratio^23^. IL-13 treatment of bat nasal^ALI^ triggered significantly increased expression of the secretory club cell marker *SCGB1A1* mRNA and a more than 1,000-fold increase in the goblet cell marker *MUC5AC*, whereas expression of the ciliated cell marker *FOXJ1* was decreased (Fig. 1j). Immunofluorescence staining of IL-13-treated bat nasal^ALI^ indicated substantially more abundant *MUC5AC*^+^ goblet cells compared to mock-treated bat nasal^ALI^ cultures (Fig. 1k). These data are consistent with a report on tracheal organoids from *Eonycteris spelaea* (cave nectar bath) and suggesting that environmental factors present in vivo may control airway goblet cell formation and maintenance^24^.

**Fig. 2:**
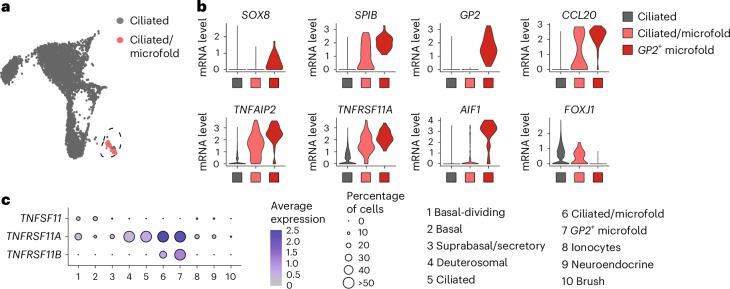
Microfold cells endogenously arise in bat bronchial organoid-derived ALI cultures. **a**, UMAP of the integrated *R. aegyptiacus*-derived nasal^ALI^ and bronchial^ALI^ scRNA-seq dataset showing the ciliated, and mixed ciliated and microfold, cell clusters. **b**, Seurat VlnPlot analysis of the microfold cell markers *SOX8*, *SPIB*, *GP2*, *TNFAPI2*, *TNFRSF11A*, *CCL20*, *TNFAPI2* and *AIF1*, and the ciliated cell marker *FOXJ1* in ciliated cell, mixed ciliated and microfold cells, and *GP2*^+^ microfold cells of *R. aegyptiacus*-derived bronchial^ALI^. The expression distribution was derived from individual cells. **c**, Seurat DotPlot showing the average expression of receptor–ligand pair markers for the RANK–RANKL signaling axis (*TNFSF11*, *TNFRSF11A* and *TNFRSF11B*) for clusters 1–10 in the *R. aegyptiacus*-derived bronchial^ALI^ scRNA-seq dataset. The dot size represents the percentage of an individual cell type expressing a given marker. Color intensity represents the average expression value. Dot size was set to a maximum percentage of 50%. (Genes expressed by more than 50% of cells have the same dot size.)

Lastly, we identified a cell cluster expressing both ciliated cell (*FOXJ1*) and microfold cell markers (*SPIB*, *CCL20* and *TNFAIP2*) in bat bronchial^ALI^ (Fig. 2a,b). Microfold cells are rare antigen-sampling cells found in the innate lymphoid tissue of the nasal and intestinal tract (Peyer’s patch) that require the RANKL–RANK signaling axis for expansion and differentiation^25^. These cells are also present at a frequency of less than 0.1% in the mouse lung^26^, making them the rarest epithelial lung cell type identified to date. A subset of cells in the ciliated and microfold cell cluster expressed high levels of the master regulators of microfold cell fate *SOX8* and *SPIB*, in addition to the bacterial uptake anchor protein encoded by *GP2*, and *RANK* (also known as *TNFRSF11A*) (Fig. 2b). These *GP2*^+^ microfold cells lacked the expression of the ciliated cell marker *FOXJ1* and comprised only 0.2% of all sequenced cells in bat bronchial^ALI^ (Extended Data Fig. 2i). Expression of *RANKL* (also known as *TNFSF11*) was largely restricted to a subset of basal cells (Fig. 2c), whereas the RANKL decoy receptor *OPG* (*TNFRSF11B*) was almost exclusively expressed in *GP2*^+^ cells (Fig. 2c), potentially limiting the continuous formation of microfold cells in bat bronchial^ALI^. In summary, our data demonstrated that organoids derived from the frozen airway tissue of *R. aegyptiacus* recapitulated multiple distinct cell lineages.

**Fig. 3:**
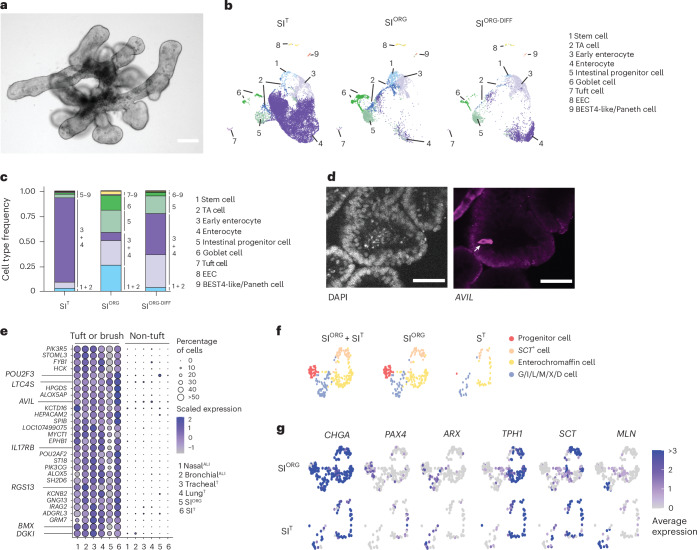
*R. aegyptiacus* SI organoids recapitulate the cellular diversity of the native bat intestinal epithelium. **a**, Representative bright-field image of *R. aegyptiacus*-derived SI^ORG^ (*n* = 3). **b**, UMAP of the integrated *R. aegyptiacus-*derived SI^T^, SI^ORG^ and differentiated SI^ORG-DIFF^ showing the individual cell types. **c**, Stacked bar plot showing the relative cell-type proportions of cell types in *R. aegyptiacus*-derived SI^T^, SI^ORG^ and differentiated SI^ORG-DIFF^. **d**, Immunofluoresence of DAPI nucleus counterstaining (left) and *AVIL* antibody staining (right) in SI^ORG^ from *R. aegyptiacus*. The arrow points to an individual *AVIL*^+^ tuft cell. **e**, Seurat DotPlot showing the scaled average expression of markers enriched in tuft and brush cells for tuft/brush, and non-tuft/brush, ECs in the *R. aegyptiacus*-derived nasal^ALI^, bronchial^ALI^, tracheal^T^, lung^T^, SI^ORG^ and SI^T^ scRNA-seq dataset. The dot size represents the percentage of an individual cell type expressing a given marker. Color intensity represents the average expression value. Dot size was set to a maximum percentage of 50%. (Genes expressed by more than 50% of cells have the same dot size.) **f**, UMAP of EEC subtypes from the integrated *R. aegyptiacus* SI^T^ + SI^ORG^ (left), SI^ORG^ (middle) or SI^T^ (right). Cells with an EEC sublineage are color-coded. **g**, Seurat FeaturePlot showing the average expression of EEC (*CHGA*) and EEC sublineage marker genes in individual cells of SI^ORG^ (top) or SI^T^ (bottom). *ARX*, differentiated G/I/L/M/X/D EECs; *TPH1*, enterochromaffin cells; *MLN*, M cells; *PAX4*, EEC progenitor cells; *SCT*, *SCT*^+^ S-like cells. The maximum color cutoff for the average expression was set to 3. Cells with an average expression of 3 or greater for a given marker have the same color. Scale bars, 50 µm.

### Bat SI^ORG^ recapitulate native SI epithelial differentiation

**Extended Data Fig. 3:**
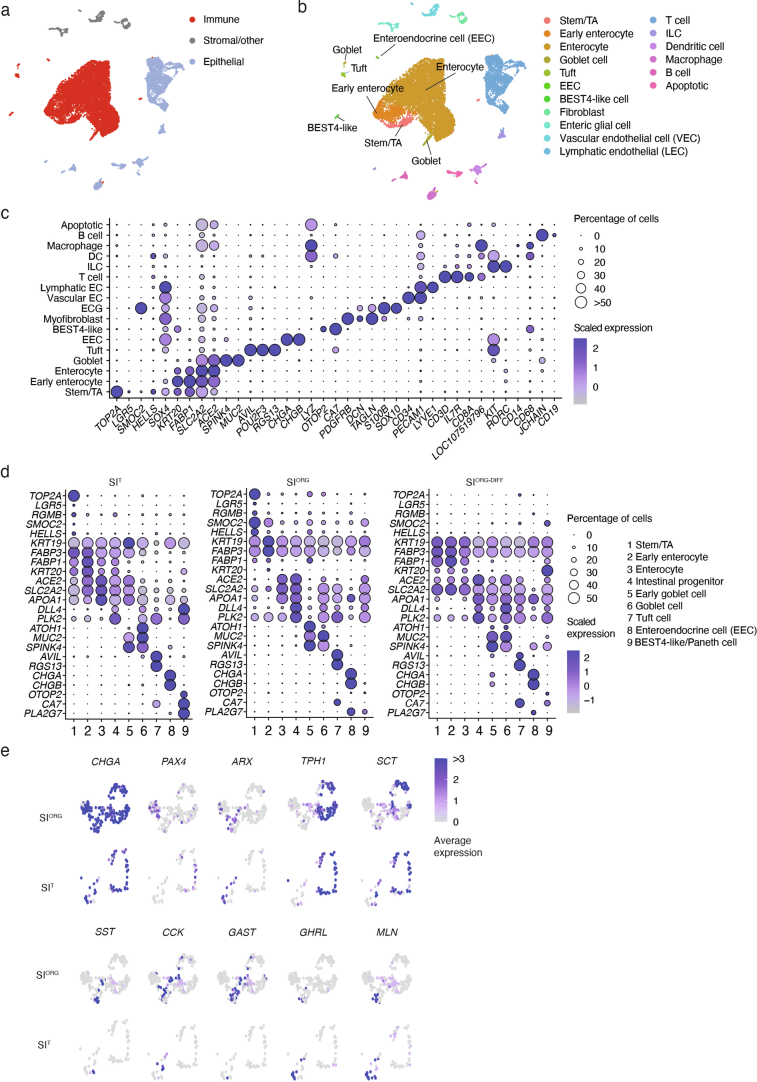
Single-cell RNA sequencing of *R. aegypticus* SI^T^ and SI^ORG^. **a**, UMAP of *R. aegyptiacus* single cell transcriptomes resolved by scRNA-seq of whole SI tissue from a captive-bred bat. Immune, stromal, and epithelial cell lineages are labeled and color-coded. **b**, UMAP of *R. aegyptiacus* SI^T^ cell types in the scRNA-seq dataset. Individual cell types are color-coded and epithelial cell types (hereafter designated as SI^T^) are additionally highlighted by text labels. **c**, Seurat *DotPlot* showing scaled average expression of marker genes for cell clusters in *R. aegyptiacus* whole SI tissue scRNA-seq dataset. Dot sizes were set to a maximum percentage of 50% (genes expressed by more than 50% of cells have the same dot size). Dot color intensity represents scaled expression. **d**, Seurat *DotPlot* showing the scaled average expression of marker genes for cell clusters in the integrated scRNA-seq data of *R. aegyptiacus* SI^T^ epithelial cells (left) and SI^ORG^ (middle) or differentiated SI^ORG^ (SI^ORG-DIFF^) (right). Dot sizes were set to a maximum percentage of 50% (genes expressed by more than 50% of cells have the same dot size). Dot color intensity represents scaled expression. **e**, Seurat *FeaturePlot* showing the average expression of Enteroendocrine cells (EECs expressing *CHGA*) and EEC sublineage marker genes in individual EECs of SI^ORG^ (top) or SI^T^ (bottom). *PAX4*, EEC progenitor; *ARX*, differentiated G/I/L/M/X/D EEC; *TPH1*, Enterochromaffin cells; *SCT*, *SCT*^+^, S-like cells; *SST*, D cells; *CCK*, I cells*; GAST*, G cells; *GHRL*, X cells; *MLN*, M cells. The maximum expression color cutoff set to 3 (cells with an average expression ≥ 3 of a given marker have the same color).

We further derived organoids from the frozen SI tissue of *R. aegyptiacus*, which could be serially passaged for at least 6 months in a niche-inspired human organoid medium^27^ (Methods). These bat SI organoids (hereafter referred to as SI^ORG^) exhibited a budded morphology with visible interspersed granular cells (Fig. 3a). To benchmark in vitro cell-type diversity, we generated an SI reference dataset by performing scRNA-seq on whole SI fragments from a captive-bred *R. aegyptiacus*, identifying distinct clusters of immune, stromal and epithelial lineages (Extended Data Fig. 3a–c and Supplementary Table 4).

ECs from the SI tissue dataset (hereafter designated SI^T^) were subsequently used for integrative analysis with bat SI organoids cultured under expansion (SI^ORG^) or differentiation (SI^ORG-DIFF^) (SI^ORG^ culture medium without WNT3A and Noggin) conditions. This analysis revealed a high concordance of cell-type diversity between SI^ORG^ and SI^T^ (Fig. 3b and Supplementary Table 4). Among the major cell types, we identified stem (*LGR5*, *SMOC2* and *CD44*) and transit-amplifying cells (*HELLS*, *PCNA*), intestinal progenitor cells (*PLK2*, *SOX4* and *DLL4*), early and mature goblet cells (*ATOH1*, *MUC2* and *SPINK4*), early enterocytes (*KRT19*^hi^, *FABP1*^hi^ and *FABP3*^hi^) and mature enterocytes (*SLC2A2*, *APOA1*, *KRT20* and *ACE2*) (Fig. 3b and Extended Data Fig. 3d). While SI^ORG^ were enriched in progenitor cell types (*LGR5*, *SMOC2*, *CD44*, *HELLS* and *SOX4*), more than 70% of all cells in SI^ORG-DIFF^ expressed markers of enterocytes (*FABP1*, *FABP3*, *SLC2A2*, *APOA1*, *KRT20* and *ACE2*), which were also the dominant cell type in SI^T^ (Fig. 3c and Extended Data Fig. 3d). scRNA-seq analyses further uncovered rare EC types in SI^ORG^ or SI^T^, namely solitary intestinal tuft cells (*POU2F3*, *AVIL* and *RGS13*) (Fig. 3d and Extended Data Fig. 3d), enteroendocrine cells (EECs) (*CHGA* and *CHGB*) and cells expressing markers of recently characterized human BEST4-like^28^ (*OTOP2* and *CA7*) or Paneth cells (*DEFA5* (also known as *LOC107504266*) and *SPIB*) (Fig. 3b,c and Extended Data Fig. 3d). Intestinal tuft cells and the related airway brush cells of bat organoids and tissue exclusively expressed a set of core genes (*AVIL*, *POU2F3*, *LT4C4S*, *RGS13*, *IRAG2*, *ALOX5AP*, *SH2D6*, *HCK*, *IL17RB*, *PIK3CG*, *BMX* and *DGKI*), distinguishing them from other non-tuft EC types present in these samples (Fig. 3e). Detailed assessment of *CHGA*^+^*CHGB*^+^ EECs in SI^ORG^ further revealed sublineages representing early EECs (*PAX4*), enterochromaffin cells (*TPH1*), *SCT*^+^ cells and various differentiated, hormone-producing EECs^29^ (M cells (*MLN*), X cells (*GHRL*), G cells (*GAST*), N cells (*NTS*), D cells (*SST*) or I cells (*CCK*)), which clustered together with EECs from SI^T^ (Fig. 3f,g and Extended Data Fig. 3e). These observations indicated that *R. aegyptiacus* SI^ORG^ closely replicated the EC type diversity observed in vivo.

### Bat organoids constitutively express innate immune effectors

**Fig. 4:**
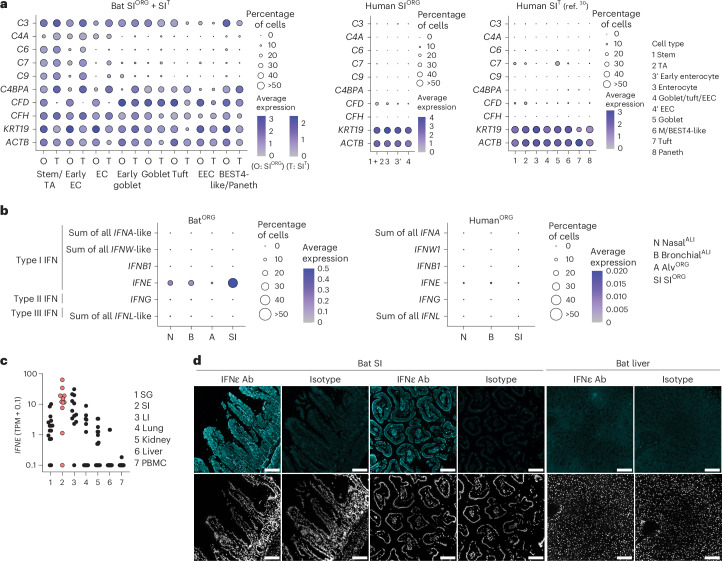
Bat organoids show heightened expression of innate immune genes, including complement system genes, IFNε and ISGs in comparison to human organoids. **a**, Seurat DotPlot showing the average expression of genes associated with the complement system and β-actin (*ACTB*) in cell types in the *R. aegyptiacus* (bat) SI^ORG^ (O) or SI^T^ (T) scRNA-seq dataset (left), human SI^ORG^ (middle) or from a published human SI^T^ scRNA-seq dataset^30^. The dot size represents the percentage of an individual cell type expressing a given marker. Color intensity represents the average expression value. Dot size was set to a maximum percentage of 50%. (Genes expressed by more than 50% of cells have the same dot size.) **b**, Seurat DotPlot showing the average expression of type I IFNα (sum of all annotated *IFNA*-like genes), IFNω (sum of all annotated *IFNW*-like genes), IFNβ (*IFNB1*), IFNε (*IFNE*), type II IFNγ (*IFNG*) and type III IFNλ (sum of all annotated *IFNL*-like genes) in the *R. aegyptiacus* (left) and human (right) nasal^ALI^ (N), bronchial^ALI^ (B), alv^ORG^ (A) or SI^ORG^ (SI) scRNA-seq dataset. Dot size represents the percentage of an individual cell type expressing a given marker. Color intensity represents the average expression value. Dot size was set to a maximum percentage of 50%. **c**, *IFNE* mRNA expression (in transcripts per million (TPM) + 0.1, pseudocount) in published bulk RNA data^32^ of *R. aegyptiacus* salivary gland (SG), SI, large intestine (LI), lung, kidney, liver and PBMCs. Each dot represents an individual bat (*n* = 11–17). Samples from the SI are highlighted in red. **d**, Representative immunofluorescence of IFNε or isotype control staining (top) and DAPI nucleus counterstaining (bottom) in *R. aegyptiacus* SI or liver sections (*n* = 3). Scale bars, 100 µm.

**Extended Data Fig. 4:**
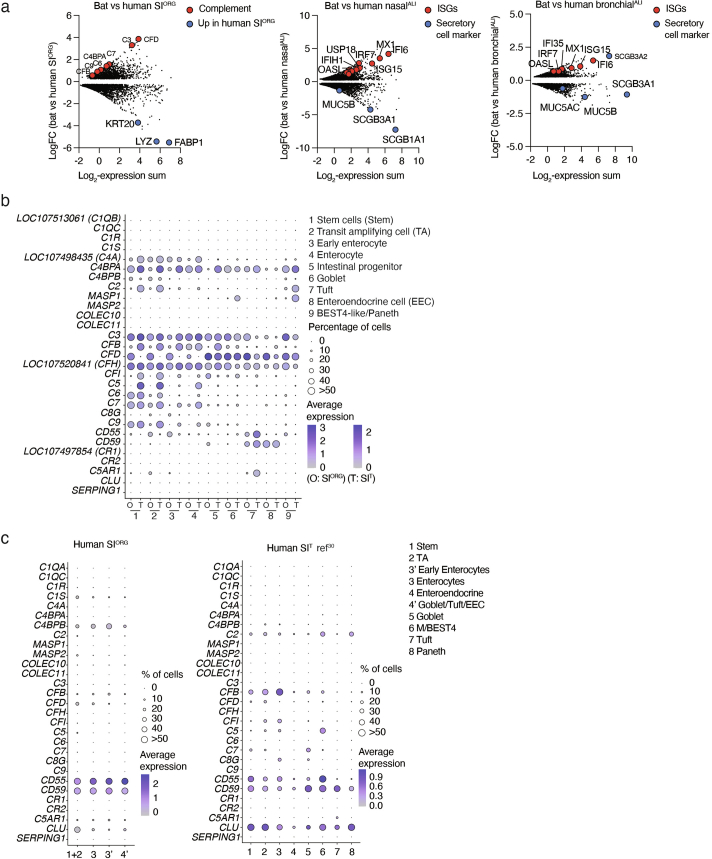
*R. aegyptiacus* SI^ORG^ and SI^T^ express heightened levels of complement system genes compared to human SI^ORG^ or human SI^T^. **a**, Seurat *FindMarker* differential gene expression analysis comparing bat to human SI^ORG^ (left), bat to human nasal^ALI^ (middle) or bat to human bronchial^ALI^, cultured side-by-side and analyzed by scRNA-seq. Each dot represents the log_2_ transformed sum of Seurat SCT normalized expression gene counts (bat + human) of differentially regulated genes (*x* axis) and the log_2_ fold changes (logFC) from the analysis between the two species (*y* axis). Positive logFC values indicate upregulated in bat organoids compared to human organoids. Selected complement system genes or interferon stimulated genes (ISGs) are labeled and colored in red. Upregulated markers in human compared to bat organoids are labeled and colored in blue. **b**, Seurat *DotPlot* showing the average expression of complement system genes in cell types in the *R. aegypticus* SI^ORG^ (O) or SI^T^ (T) scRNA-seq dataset. Dot size, the percentage of an individual cell type expressing a given marker. Color intensity, the average expression value. Dot sizes were set to a maximum percentage of 50% (genes expressed by more than 50% of cells have the same dot size). **c**, same as in **(b)** but in the human SI^ORG^ scRNA-seq (left) or in a published human SI^T^ scRNA-seq datasets (ref. ^30^).

Next, we performed comparative RNA expression analysis of bat SI^ORG^, nasal^ALI^ or bronchial^ALI^ to human nasal^ALI^, bronchial^ALI^ or SI^ORG^, prepared in parallel under identical conditions to bat counterparts. We detected a heightened expression of genes with innate immune effector functions (IFN-stimulated genes (ISGs) and complement genes) in bat over human organoid models (Extended Data Fig. 4a). For example, bat SI^ORG^ exhibited high and exclusive expression of several genes associated with the classical and alternative complement system (that is, *C2*, *C3*, *C6*, *C7*, *C9*, *C4BPA*, *CFD* and *CFH* (also known as *LOC107520841*)) compared to human SI^ORG^ or to a public scRNA-seq dataset from the human SI epithelium^30^ (Fig. 4a and Extended Data Fig. 4b,c), which is consistent with a report on the expression of complement genes in barrier tissues in *R. aegyptiacus*^5^.

**Extended Data Fig. 5:**
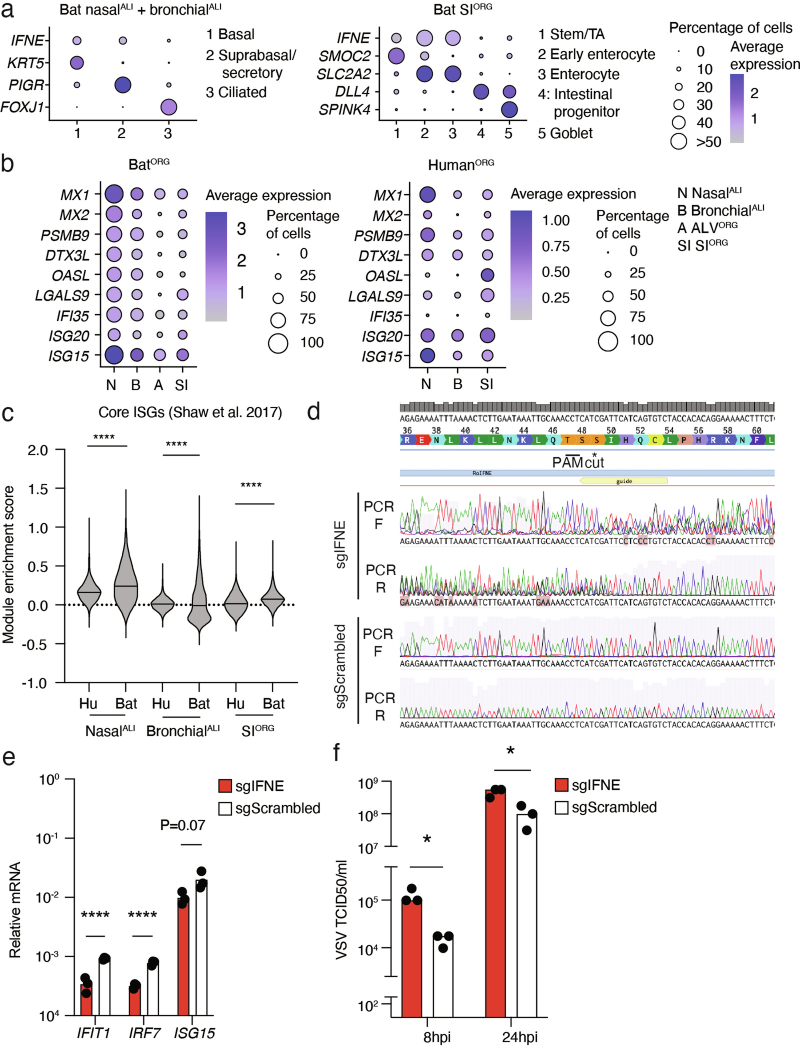
Comparison of *IFNE* or ISG expression in *R. aegyptiacus* and human organoids, and genetic perturbation of *IFNE* in bat SI^ORG^. **a**, Seurat *DotPlot* showing the average expression of *IFNE* and cell type-specific marker genes in the integrated bat nasal^ALI^ + bronchial^ALI^ (left), or bat SI^ORG^ scRNA-seq dataset. Dot size, the percentage of an individual cell type expressing a given marker. Color intensity, the average expression value. Dot sizes were set to a maximum percentage of 50% (genes expressed by more than 50% of cells have the same dot size). **b**, Seurat *DotPlot* showing the average expression of ISGs in the bat nasal^ALI^, bronchial^ALI^, alv^ORG^ or SI^ORG^ scRNA-seq dataset (left), or human nasal^ALI^, bronchial^ALI^ or SI^ORG^ scRNA-seq dataset (right). Dot size, the percentage of an individual cell type expressing a given marker. Color intensity, the average expression value. **c**, Violin plots showing the ISG module enrichment score distribution for conserved ISGs (ref. 33) in scRNA-seq of bat or human nasal^ALI^, bronchial^ALI^ or SI^ORG^. The distribution was derived from the enrichment scores of individual cells. Median shown as solid line. A positive score indicates enrichment of ISGs in a culture model. Two-sided Mann-Whitney tests were performed to compare human to bat nasal^ALI^, bronchial^ALI^ or SI^ORG^ (****: P-value < 0.0001). **d**, Sanger sequencing trace of PCR amplicons spanning the bat *IFNE* gene in bat SI^ORG^ expressing Cas9 and a guide RNA targeting *IFNE* (sgIFNE) or a non-targeting control guide RNA (sgScrambled). The guide RNA spacer sequence and expected cut site is shown above. **e**, RT-qPCR analysis of ISGs, normalized to *EEF1A1* (2^–ΔCT^), in Cas9-sgRNA expressing bat SI^ORG^ (n = 3). Two-sided unpaired Student’s t-tests were performed to compare ISG expression between sgIFNE-SI^ORG^ and sgScrambled-SI^ORG^ (**** P < 0.0001). **f**, VSV titer measured from the culture supernatant of infected sgIFNE (n = 3) or sgScrambled bat SI^ORG^ (n = 3) after 8 or 24 hours post infection (hpi). The titer was derived from TCID50 assays performed in VeroE6 cells with five replicates per sample supernatant.

We also observed high expression of IFNε mRNA (encoded by *IFNE*) in bat SI^ORG^, nasal^ALI^ or bronchial^ALI^ compared to human counterparts (Fig. 4b). IFNε is a noncanonical type I IFN with antiviral and immune-regulatory functions, typically restricted to the female reproductive tract in humans and mice^31^. *IFNE* was the only IFN gene (among type I *IFNA*-like subtypes, *IFNB1*, *IFNW*-like subtypes, type II *IFNG* or type III *IFNL*-like) expressed at robustly detectable levels (more than 5% in any given organoid model) in the bat SI^ORG^, nasal^ALI^ or bronchial^ALI^ scRNA-seq dataset (Fig. 4b). Increased expression of *IFNE* was particularly prominent in the enterocytes of SI^ORG^ and the basal cells of nasal^ALI^ or bronchial^ALI^ (Extended Data Fig. 5a). The highest expression of *IFNE* was also detected in the intestine in a public bulk RNA-seq dataset^32^ that evaluated several nonreproductive tissues (salivary gland, intestine, liver, lung, spleen and peripheral blood mononuclear cells (PBMCs)) of *R. aegyptiacus* (Fig. 4c). Immunofluorescence analysis in *R. aegyptiacus* tissue sections showed strong IFNε staining along the SI villi, but not in the liver or isotype controls (Fig. 4d). The basal expression of *IFNE* was associated with a heightened basal expression of conserved ISGs^33^ in nasal^ALI^, bronchial^ALI^ or SI^ORG^ (for example, *MX1*, *MX2*, *IRF7*, *PSMB9*, *DTX3L*, *OASL*, *LGALS9* and *ISG15*) (Extended Data Fig. 5b), suggesting a potential role for IFNε in constitutive antiviral immunity, consistent with its reported function in mouse reproductive tissue^31^. Of note, we observed higher mRNA expression of ISGs in both human and bat nasal^ALI^ cultures compared to bronchial^ALI^ or SI^ORG^ (Extended Data Fig. 5c), indicating that additional factors beyond IFNε contribute to this effect. Nevertheless, bat nasal^ALI^, bronchial^ALI^ or SI^ORG^ exhibited significantly higher expression of conserved ISGs compared to their human counterparts cultured in parallel (Extended Data Fig. 5c).

To investigate the role of IFNε in bat epithelial antiviral immunity, we disrupted the *IFNE* gene in bat SI^ORG^ using lentivirus delivery for CRISPR–Cas9 and single-guide RNAs (sg RNAs) (Extended Data Fig. 5d). Quantitative PCR with reverse transcription (RT–qPCR) analysis of selected ISGs (*IFIT1* (also known as *LOC107501624*), *IRF7* and *ISG15*) revealed a modest but significant reduction in the mRNA expression of these genes in IFNε-perturbed organoids (sgIFNE) compared to organoids transduced with a nontargeting control guide RNA (sgScrambled) (Extended Data Fig. 5e). Vesicular stomatitis virus encoding the enhanced green fluorescent protein (VSV-eGFP), which is sensitive to bat IFNs^4^, replicated to higher titers in infected sgIFNE-SI^ORG^ compared to sgScrambled-SI^ORG^ (Extended Data Fig. 5f), indicating increased susceptibility to viral infection. Together, these findings revealed differences in gene expression between bat and human respiratory and intestinal ECs, particularly in genes linked to constitutive innate immunity, which may contribute to enhanced resistance to pathogens at bat epithelial surfaces.

### MARV infection triggers an IFN response in bat organoids

**Fig. 5:**
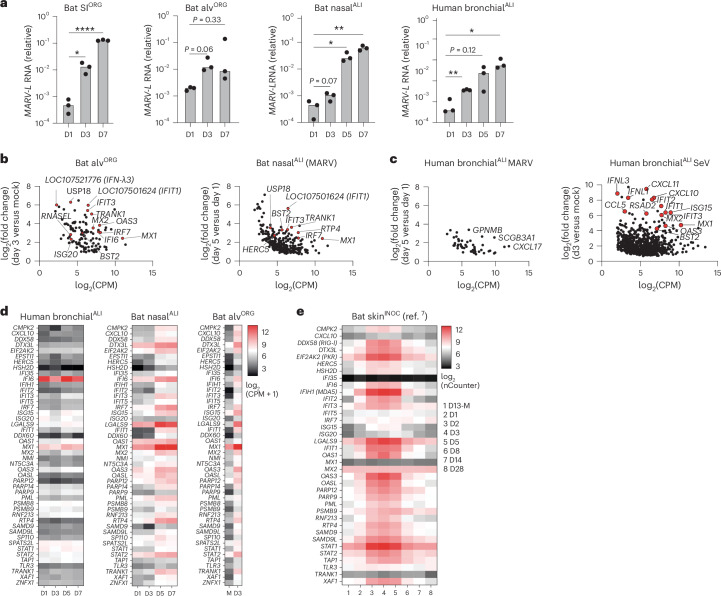
MARV infection in bat organoids triggers an IFN response. **a**, RT–qPCR analysis of intracellular *MARV-L* expression (normalized to *EEF1A1*, 2^-ΔCt^) in bat SI^ORG^(*n* = 3), alv^ORG^(*n* = 3), nasal^ALI^ (*n* = 3) or human bronchial^ALI^ (*n* = 3) infected with MARV (Musoke strain) at an estimated multiplicity of infection (MOI) of 0.5–1 (50,000 plaque forming units (PFU) for bat SI^ORG^ and alv^ORG^; 100,000 PFU for bat nasal^ALI^ or human bronchial^ALI^) from day 1 to day 7 (D1–D7) after infection. A two-sided Student’s *t*-test was used to compare the average intracellular *MARV-L* mRNA level at each time point to the first analyzed time point (D1) for each condition. *P* > 0.05, no significant difference, *P < 0.05, **P < 0.01,, ****P < 0.0001. **b**, EdgeR differential gene expression analysis of bat alv^ORG^ (*n* = 3) infected with MARV for 72 h compared to mock-infected (left) or bat nasal^ALI^ (*n* = 3) at day 5 after infection compared to day 1 after infection with MARV (right). Each dot represents the expression (in log_2_-transformed counts per million (CPM)) and log_2_-transformed fold change of a differentially expressed gene (DEG) between two comparisons. Selected ISGs are highlighted in red and labeled. **c**, Human bronchial^ALI^ (*n* = 3) at day 5 after infection compared to day 1 after infection with MARV (left) or 72 h after infection with SeV compared to mock infection (right). Each dot represents the expression (in log_2_(CPM)) and log_2_(fold change) of a DEG between two comparisons. Selected genes are labeled and highlighted in red for ISGs. **d**, Heatmap showing normalized gene expression (log_2_(CPM + 1)) from bulk RNA-seq for ISGs in human bronchial^ALI^ (left), bat nasal^ALI^ (middle) or bat alv^ORG^ (right) from day 1 to day 7 (D1–D7) after infection with MARV or day 3 in mock infection (M). Each mRNA value represents the average of three biological replicates. **e**, Heatmap displaying log_2_-normalized gene expression of ISGs from reanalyzed public nCounter NanoString data of skin samples (inoculation site) from *R. aegyptiacus*^7^ (bat skin^INOC^, *n* = 4–6) from day 1 to day 28 (D1–D28) after infection with MARV or day 13 in mock infection (D13-M). Each mRNA value represents the average of 4–6 bats in the published dataset.

**Extended Data Fig. 6:**
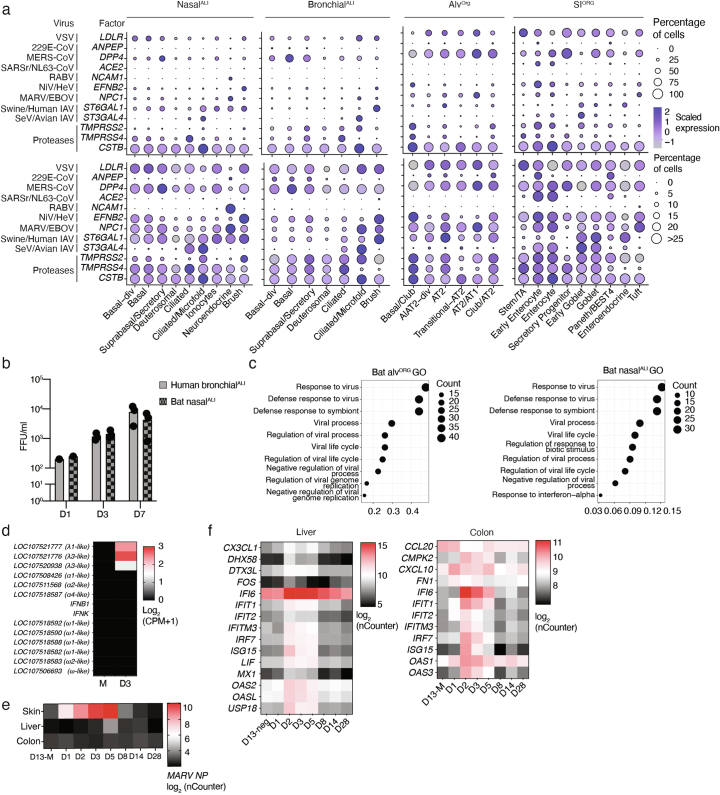
Characterization of virus entry factor expression and antiviral responses to MARV infection in *R. aegyptiacus* organoids and animals. **a**, Seurat *DotPlot* analysis showing the average expression of virus entry factors of cell types in the bat nasal^ALI^, bronchial^ALI^, alv^ORG^ or SI^ORG^ scRNA-seq dataset. Dot size, the percentage of an individual cell type expressing a given marker. Color intensity, the scaled average expression value. Dot sizes were set to a maximum percentage of 50% (top) or 25% (bottom). Genes expressed by more than 50% or 25% of cells have the same dot size. **b**, MARV (Musoke) titer (in focus forming units per ml (FFU/ml)) measured from combined solutions of apical washes and basal chamber medium of infected human Bronchial^ALI^ (n = 3) or bat Nasal^ALI^ (n = 3) after one-, three- or seven-days post infection. **c**, Gene ontology (GO) enrichment analysis, performed using *clusterProfiler*, revealed biological processes significantly enriched among genes upregulated in MARV-infected vs. mock-infected bat alv^ORG^, or in bat nasal^ALI^ at day 5 vs. day 1 post-infection. The top 10 enriched biological processes are shown. The *x* axis indicates the ratio of enriched to total genes within each GO term. Dot size reflects the number of enriched genes per pathway. **d**, Heatmap showing normalized gene expression (log_2_-counts per million + 1) from bulk RNA-seq for IFN subtypes in MARV-infected or mock-infected bat alv^ORG^. Each mRNA value represents the average of three biological replicates. Days post-infection are indicated (D3: day 3 post infection, M: day 3 mock-infected). **e**, Heatmap displaying log₂-normalized gene expression of *MARV NP* from reanalyzed public nCounter NanoString data of skin, liver, and colon samples from MARV-infected *R. aegyptiacus* (n = 4–6). Each mRNA value represents the average of four to six animals present in the dataset. Days post-infection are indicated (D1–D28, D13-M: day 13 mock-infected). **f**, Heatmap displaying log₂-normalized gene expression of ISGs from reanalyzed public nCounter NanoString data of liver and colon samples from MARV-infected *R. aegyptiacus* (n = 4–6). Each mRNA value represents the average of four to six animals present in the dataset. Days post-infection are indicated (D1–D28, D13-M: day 13 mock-infected).

Organoids hold great potential as in vitro models to investigate host–virus interactions at near physiological levels. scRNA-seq analysis of bat nasal^ALI^, bronchial^ALI^, alv^ORG^ or SI^ORG^ revealed the expression of viral entry factors for multiple zoonotic viruses of human concern, including *NPC1*, the entry receptor for Ebola virus (EBOV) and MARV, *ACE2* (for SARS-CoV-1 and SARS-CoV-2), *DPP4* (for Middle East respiratory syndrome coronavirus (MERS-CoV)), *EFNB2* (for the Hendra and Nipah viruses) and *TMPRSS2*/*TMPRSS4* (serine proteases used by SARS-CoV-2, MERS-CoV or the influenza A virus (IAV) subtypes H1N1 and H7N9) (Extended Data Fig. 6a). Given the disparate disease outcomes of MARV infection in *R. aegyptiacus* bats^7^ compared to humans^34^, together with a suspected infection route via mucosal surfaces in these species^35^, we investigated the susceptibility and cellular responses to MARV infection in bat SI^ORG^, nasal^ALI^ or alv^ORG^, and well-differentiated human bronchial^ALI^ cultures. RT–qPCR indicated a tenfold to 1,000-fold increase in the *MARV-L* RNA (Musoke isolate) in SI^ORG^, nasal^ALI^ or alv^ORG^ between day 3 and day 7 after infection compared to day 1 (Fig. 5a), while human bronchial^ALI^ cultures infected with MARV also displayed a significant increase in *MARV-L* RNA expression over a 7-day time course experiment (Fig. 5a), indicating that differentiated *R. aegyptiacus* and human ECs supported entry and replication of MARV. Notably, we detected higher release of infectious MARV in human bronchial^ALI^ compared to bat nasal^ALI^ at day 5 and day 7 after infection (Fig. 5a and Extended Data Fig. 6b). Next, we used pooled 3′-end sample-barcoded bulk RNA-seq (hereafter bulk RNA-seq) to investigate the genome-wide transcriptional landscape in nasal^ALI^, alv^ORG^ or human bronchial^ALI^ infected with MARV (Fig. 5b–d and Supplementary Table 5). We found a significant temporal induction of ISGs (for example, *MX1*, *RTP4*, *IRF7*, *USP18*, *TRANK1*) in alv^ORG^ at day 3 after infection compared to mock-infected samples or nasal^ALI^ at day 5 and day 7 compared to day 1 after infection (Fig. 5b,d and Supplementary Table 5). Gene Ontology (GO) enrichment analysis of upregulated genes in MARV-infected bat alv^ORG^ or nasal^ALI^ revealed a strong enrichment of pathways associated with viral defense and IFN signaling (for example, defense response to virus (accession GO:0051607), negative regulation of viral process (accession GO:0048525), positive regulation of type I IFN production (accession GO:0032481)) (Extended Data Fig. 6c). Among IFNs, we detected the mRNA of an *IFNL1*-like gene (also known as *LOC107521777*) and two distinct *IFNL3*-like genes (also known as *LOC107521776* or *LOC107520938*) as the most robustly induced IFNs in MARV-infected versus uninfected bat alv^ORG^ (Extended Data Fig. 6d). By contrast, MARV-infected human bronchial^ALI^ showed little to no significant ISG induction (Fig. 5c,d and Supplementary Table 5), but triggered a strong IFN response (for example, *IFNL1*, *IFNL3*, *IFIT3*, *MX1*, *RSAD2*, *BST2* and *ISG15*) when challenged with murine Sendai virus (SeV).

The induction of ISGs in MARV-infected bat nasal^ALI^ or alv^ORG^, but not human bronchial^ALI^, was also observed in reanalyzed public mRNA expression data from *R. aegyptiacus*^7^ infected subcutaneously with MARV (Fig. 5e). There, ISG expression peaked at day 3–5 after infection at the skin inoculation site and to a lesser extent in other tissues, such as the liver and colon, where viral *MARV-NP* mRNA levels remained low, before returning to baseline by day 28 (Fig. 5e and Extended Data Fig. 6e,f). Thus, MARV-infected bat respiratory epithelial organoids recapitulated antiviral gene expression changes at barrier tissues in MARV-infected bats, contrasting with the blunted IFN responses observed in MARV-infected differentiated human airway EC cultures.

### Bat organoids predominantly induce type III IFNs after virus infection

**Fig. 6:**
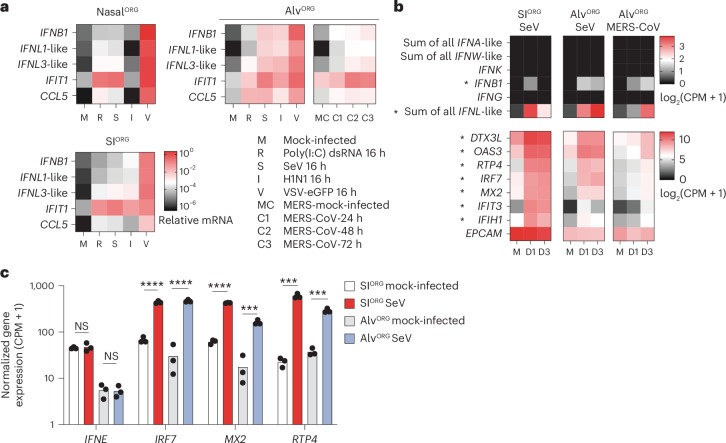
Type III IFNs are predominantly induced in bat organoids upon zoonotic virus infection. **a**, RT–qPCR analysis of type I *IFNB1*, type III *IFNL1*-like (*LOC107521777*), *IFNL3*-like (*LOC107521776*), *IFIT1* (*LOC107501624*) and *CCL5* (normalized to *EEF1A1*, 2^-ΔCt^) in virus-infected (SeV, H1N1, VSV-eGFP or MERS-CoV), poly(I:C)-transfected or mock-treated bat nasal^ORG^, SI^ORG^ or alv^ORG^. Each mRNA value represents the average of three biological replicates. SeV (S), H1N1 (I) and VSV (V) infections were performed at an MOI of 0.5 for 16 h; poly(I:C) (R) was transfected at 1 µg ml^−1^ for 16 h and MERS-CoV infections were conducted for 24 (C1), 48 (C2) or 72 h (C3) or mock (MC). **b**, Heatmap showing normalized gene expression (log_2_(CPM + 1)) from bulk RNA-seq in SeV-infected bat SI^ORG^ (left), SeV-infected bat alv^ORG^ (middle) or MERS-CoV-infected bat alv^ORG^ (right) for *IFNA*-like (sum of all *IFNA*-like genes), *IFNW*-like (sum of all *IFNW*-like genes), *IFNK*, *IFNB1*, *IFNG*, *IFNL*-like (sum of all *IFNL*-like genes) (top) and ISGs or the epithelial marker *EPCAM* (bottom) at D1–D3 after infection. Each mRNA value represents the average of three biological replicates. The single asterisk indicates IFN or ISGs expressed in at least two of three biological replicates. **c**, Normalized gene expression (CPM + 1) from bulk RNA-seq for *IFNE* and the ISGs *IRF7*, *MX2* or *RTP4* in SeV-infected or mock-infected bat SI^ORG^ (*n* = 3) or bat alv^ORG^ (*n* = 3). Biological replicates are represented by the dots, with the bar height indicating the average expression. A two-sided Student’s *t*-test was used to compare the average mRNA expression levels of each gene in SeV-infected organoids versus uninfected organoids at day 3 after infection. NS, not significant; ***P < 0.001, ****P < 0.0001.

Next, we extended our findings on the antiviral responses to MARV infection in bat organoids to other zoonotic RNA viruses not naturally found in *R. aegyptiacus*. RT–qPCR analysis of bat nasal^ORG^, alv^ORG^ or SI^ORG^ infected with murine SeV, VSV-eGFP, MERS-CoV or human IAV (H1N1, cell-culture-adapted A/WS/33 strain), showed the induction of *IFNL1*-like (*LOC107521777*), *IFNL3*-like (*LOC107521776* and *LOC107520938*) and *IFNB1*, as well as downstream ISGs, such as *IFIT1* and *CCL5* (Fig. 6a). Poly(I:C) double-stranded RNA (dsRNA) treatment of nasal^ORG^, alv^ORG^ and SI^ORG^ induced these genes to similar levels compared to viral infection (Fig. 6a).

To broaden our analysis from selected genes to all annotated IFNs and ISGs, we performed bulk RNA-seq of SeV-infected bat alv^ORG^ or SI^ORG^, and MERS-CoV-infected alv^ORG^. We found that, in addition to a broad induction of ISGs (for example, *DTX3L*, *OAS3*, *RTP4*, *IRF7*, *MX2*, *IFIT3*, *IFIH1*), only *IFNB1* and, more robustly, *IFNL1*-like and *IFNL3*-like were induced in virus-infected organoids at 1 and 3 days after infection compared to the uninfected controls (Fig. 6b and Supplementary Table 5). *IFNE*, which was constitutively expressed, was not further increased by SeV infection in bat SI^ORG^ or alv^ORG^ (Fig. 6c). This is consistent with reports in mice, where IFNε is not induced by classical pattern recognition receptor ligands associated with RNA viruses^31^. These observations indicated that IFNλ genes were the predominant and most robustly induced IFN produced after viral infection in *R. aegyptiacus* ECs.

### IFNλs drive robust antiviral gene responses in bat organoids

**Fig. 7:**
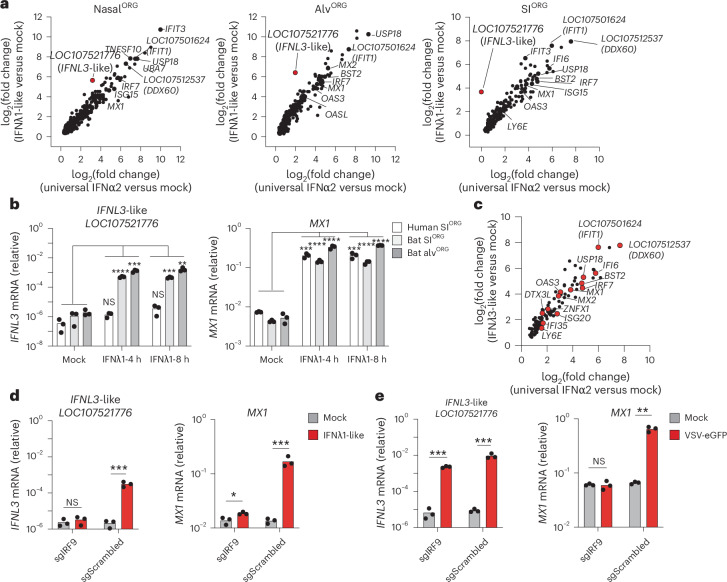
Bat type III IFNλ drive self-amplified antiviral responses in bat organoids. **a**, Scatter plots showing the log_2_-transformed fold changes in gene expression from bulk RNA-seq comparing nasal^ORG^ (left), alv^ORG^ (middle) or SI^ORG^ (right) treated with universal IFNα2 (*x* axis) or IFNλ1-like (*y* axis) to mock-treated controls after 8 h. Individual ISGs are highlighted. *IFNL3*-like (*LOC107521776*) is highlighted in red. **b**, RT–qPCR analysis of *IFNL3*-like (*LOC107521776*) in bat SI^ORG^ or alv^ORG^; *IFNL3* in human SI^ORG^) normalized to *EEF1A1* (2^−Δ*C*t^) after treatment with bat IFNλ1-like (bat organoids) or human IFNλ1 (human SI^ORG^) for 4 and 8 h. A two-sided Student’s *t*-test was used to compare *IFNL3*-like mRNA levels at 4 or 8 h after treatment to mock-treated controls. **c**, Scatter plot showing the log_2_-transformed fold changes in gene expression from bulk RNA-seq comparing SI^ORG^ treated with universal IFNα2 (*x* axis) or IFNλ3-like (derived from *LOC107521776*) (*y* axis) to mock-treated controls after 8 h. Individual ISGs are highlighted in red. **d**, RT–qPCR analysis of *IFNL3*-like (*LOC107521776*) or *MX1* (normalized to *EEF1A1*, 2^−^^Δ*C*t^) in bat SI^ORG^ engineered with Cas9 and IRF9 targeting (sgIRF9) or control (sgScrambled) RNA, treated with or without bat IFNλ1-like for 8 h. A two-sided Student’s *t*-test was used to compare *IFNL3*-like mRNA levels between sgIRF9 and sgScrambled SI^ORG^ at 8 h after treatment. **e**, As in **d** but comparing sgIRF9 and sgScrambled SI^ORG^ after infection with VSV-eGFP at an MOI of 0.05 for 72 h. *P < 0.05, **P < 0.01, ***P < 0.001, ****P < 0.0001.

**Extended Data Fig. 7:**
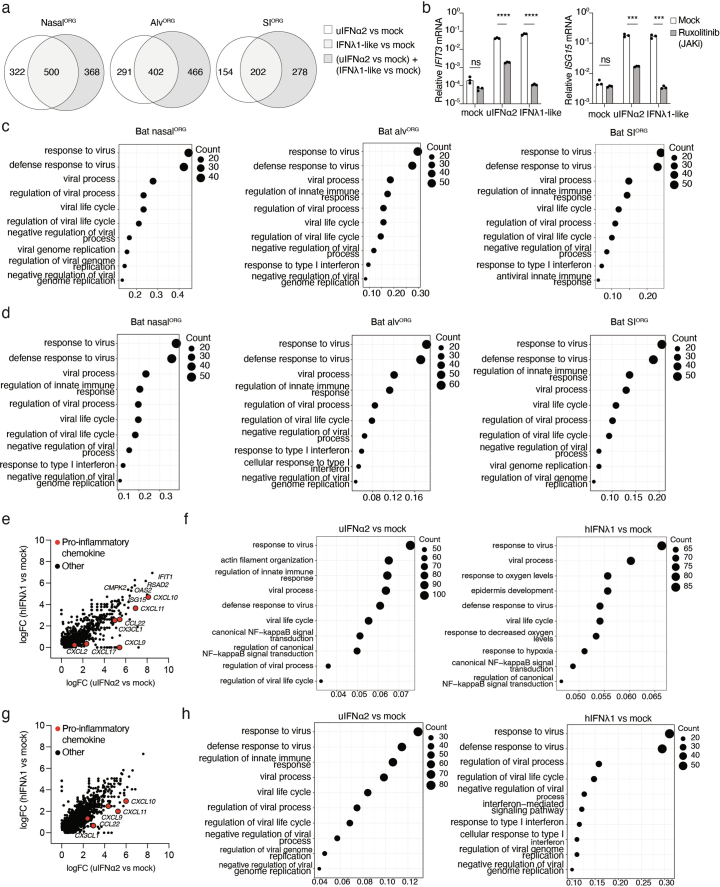
Type I/III IFNs drive antiviral gene responses in bat and human organoids. **a**, Venn diagrams (generated using *eulerr.co*) showing the number of significantly upregulated genes determined by bulk RNA-seq in bat nasal^ORG^, alv^ORG^, or SI^ORG^ (n = 3 each) following treatment with universal IFNα2 (uIFNα2) or IFNλ1-like compared to mock-treated. The total number of upregulated genes per treatment are indicated. Overlapping regions represent genes commonly induced by both IFNs. **b**, RT-qPCR analysis of *IFIT3* and *ISG15* expression, normalized to *EEF1A1* (2^–ΔCT^), in bat nasal^ORG^ (n = 3) treated with uIFNα2 or IFNλ1-like for 8 h, with or without ruxolitinib (n = 3). Two-sided unpaired Student’s t-tests were used to compare mRNA levels between ruxolitinib-treated and mock-treated, interferon-stimulated nasal^ORG^. (ns: not significant; *** P < 0.001, **** P < 0.0001). **c**, Gene ontology (GO) enrichment analysis of bulk RNA-seq data, performed with *clusterProfiler*, revealed biological processes significantly enriched among genes upregulated in uIFNα2 vs. mock-infected bat nasal^ORG^, alv^ORG^, or SI^ORG^ after 8 h (n = 3 each). The top 10 enriched biological processes are shown. The *x* axis indicates the ratio of enriched to total genes within each GO term. Dot size reflects the number of enriched genes per pathway. **d**, Same as in **(c)** but for IFNλ1-like vs. mock-treated bat nasal^ORG^, alv^ORG^, or SI^ORG^. **e**, Scatter plot showing log_2_-fold changes in gene expression from bulk RNA-seq comparing human SI^ORG^ treated with uIFNα2 (*x* axis) or IFNλ1-like (*y* axis) to mock-treated controls after 8 h (n = 3). Individual ISGs and selected pro-inflammatory cytokines (red) are highlighted and labeled. **f**, same as in **(c)** but for uIFNα2 (left) or IFNλ1 (right) vs. mock-treated human SI^ORG^. **g**, Same as in **(e)** but for human bronchial^ALI^ (n = 3). **h**, Same as in **(f)** but for GO enrichment analysis of uIFNα2 (left) or IFNλ1 (right) vs. mock-treated human bronchial^ALI^.

**Extended Data Fig. 8:**
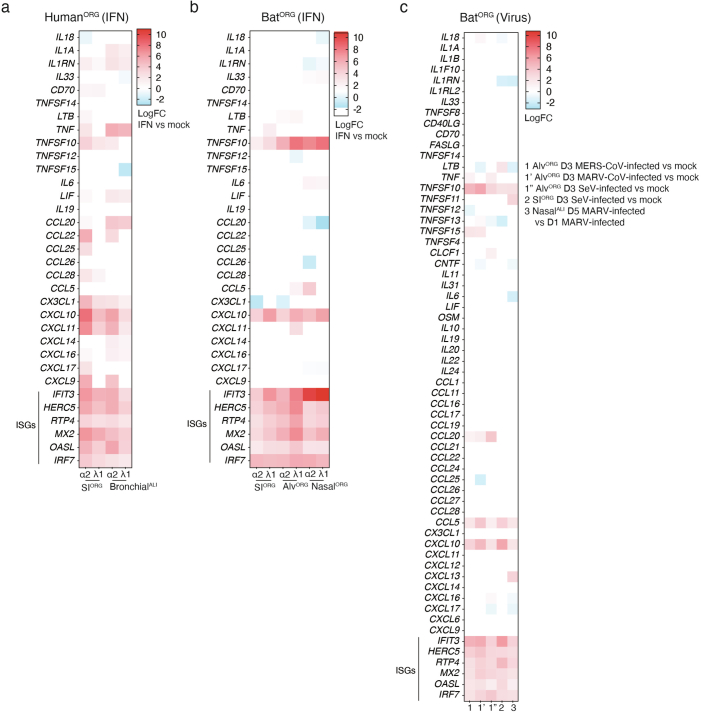
Pro-inflammatory genes are not broadly induced in bat organoids treated with IFN or infected with RNA viruses. **a**, Heatmap showing gene expression log_2_-fold changes (from *EdgeR* bulk RNA-seq differential gene expression analysis) of selected pro-inflammatory genes and selected ISGs in universal IFNα2 (uIFNα2) or IFNλ1 vs. mock-treated human SI^ORG^ or human Bronchial^ALI^ (n = 3 each). **b**, Same as for **(a)** but for IFN-treated bat SI^ORG^ (n = 3), bat alv^ORG^ (n = 3), or bat nasal^ORG^ (n = 3). **c**, Same as for **(a)** but for MARV-, MERS-CoV- or SeV-infected bat alv^ORG^ (n = 3 each), SeV-infected bat SI^ORG^ (n = 3), or MARV-infected bat nasal^ALI^ (n = 3). Comparisons used for calculation of *edgeR* log_2_-fold change are indicated in the legend.

In mice and humans, type I and III IFNs induce a largely overlapping set of genes that perform common antiviral functions^36^. To compare type III IFN-mediated and type I IFN-mediated gene induction in *R. aegyptiacus* ECs, we performed bulk RNA-seq on bat nasal^ORG^, alv^ORG^, or SI^ORG^ treated for 8 h with recombinant homemade *R. aegyptiacus* type III IFNλ1-like (see Methods), universal type I IFNα2 or PBS (mock-treated). Universal type I IFNα2 is a recombinant IFNα hybrid protein that exhibits bioactivity across multiple species^33^, including *R. aegyptiacus*^37^. In parallel, we treated human SI^ORG^ or bronchial^ALI^ with recombinant human IFNλ1 or universal type I IFNα2 for 8 h to establish a cross-species reference dataset. Bulk RNA-seq revealed significant induction of classical ISGs (for example, *ADAR*, *IFIT1*, *IFIT2*, *IFIT3*, *IFI6*, *IFIH1*, *OAS3*, *OASL*, *IRF7*, *USP18* and *ZNFX1*) in bat nasal^ORG^, alv^ORG^ or SI^ORG^ incubated with universal IFNα2 or bat IFNλ1-like compared to mock-treated organoids (Fig. 7a and Supplementary Table 6). We identified 124 genes that were significantly upregulated by both IFN treatments in all bat organoid models (for example, *ADAR*, *IRF7*, *ISG15*, *LGALS9*, *MX2*, *PSMB9* and *ZNFX1*) (Supplementary Table 6). Notably, IFNλ1-like treatment significantly upregulated more genes relative to mock treatment than universal IFNα2, across all bat organoid models (Extended Data Fig. 7a and Supplementary Table 6). Co-treatment with the Janus kinase (JAK) inhibitor ruxolitinib abolished the induction of the ISGs *IFIT3* or ISG15 by universal IFNα2 or bat IFNλ1-like in nasal^ORG^ (Extended Data Fig. 7b), indicating that both type I and type III IFNs signal through the JAK–signal transducer and activator of transcription (STAT) signaling pathway in *R. aegyptiacus* ECs. GO terms related to antiviral defense and IFN responses (defense response to virus (accession GO:0051607), negative regulation of viral process (accession GO:0048525), response to type I IFN (accession GO:0034340)) were the top enriched biological processes in response to both bat IFNλ1-like and universal IFNα2 treatment (Extended Data Fig. 7c,d). Similarly, universal IFNα2 or human IFNλ1 stimulation of human SI^ORG^ or human bronchial^ALI^ induced the expression of antiviral response genes (for example, *IFIT1*, *IFIT2*, *IFIT3*, *OAS1, OAS2*, *OAS3*, *IRF7* and *RSAD2*) (Extended Data Fig. 7e–h and Supplementary Table 6). However, we observed a stronger induction of pro-inflammatory chemokines (for example, *CXCL1*, *CXCL2*, *CXCL9*, *CXCL11*, *CX3CL1* and *CCL22*) in human SI^ORG^ or bronchial^ALI^ treated with universal IFNα2 compared to human IFNλ1, aligning with previous results published in mice^8^ (Extended Data Fig. 7e–h and Extended Data Fig. 8a). By contrast, universal IFNα2 or bat IFNλ1-like-treatment or virus-infection of bat nasal^ORG^ (MARV), alv^ORG^ (MARV, MERS, SeV) or SI^ORG^ (SeV), resulted in minimal or no changes in the expression of these pro-inflammatory genes (Extended Data Fig. 8b,c).

**Extended Data Fig. 9:**
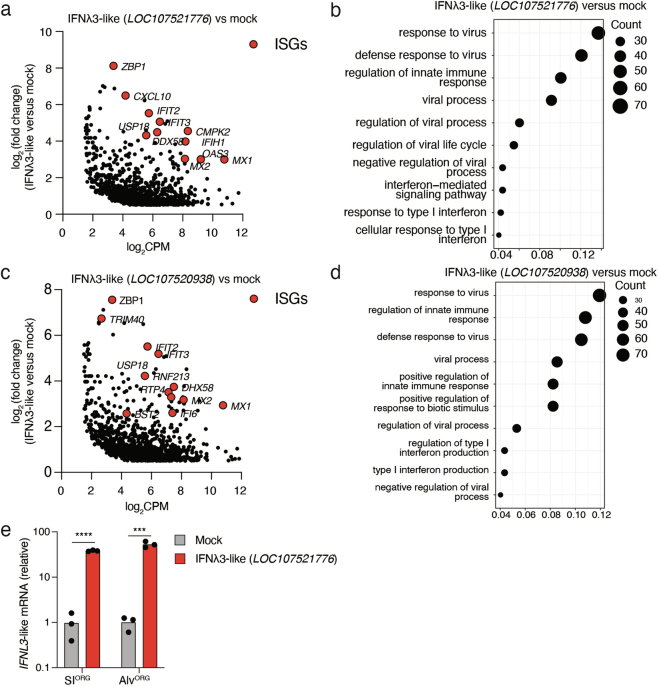
Bat type III IFNλ3-like drive self-regulated antiviral responses in bat SI^ORG^. **a**, *EdgeR* differential gene expression analysis of bat SI^ORG^ (n = 3) treated with IFNλ3-like (*LOC107521776*) (dose equivalent to 1000 U/ml universal IFNα2) for 8 h compared to mock-treated (n = 3). Each dot represents the average expression (in log_2_-count per million, *x* axis) and log_2_-fold change (*y* axis) of a differentially expressed gene between IFNλ3-like vs. mock-treated SI^ORG^. Selected ISGs are highlighted in red and labeled. **b**, Gene ontology (GO) enrichment analysis of bulk RNA-seq data, performed with *clusterProfiler*, revealed biological processes significantly enriched among genes upregulated in IFNλ3-like vs. mock-infected bat SI^ORG^ after 8 h (n = 3 each). The top 10 enriched biological processes are shown. The x-axis indicates the ratio of enriched to total genes within each GO term. Dot size reflects the number of enriched genes per pathway. **c**, Same as in **(a)** but for IFNλ3-like (*LOC107520938*) vs. mock-treated SI^ORG^ (n = 3). **d**, Same as in **(b)** but for IFNλ3-like (*LOC107520938*) vs. mock-treated SI^ORG^. **e**, RT–qPCR analysis of *IFNL3*-like (*LOC107521776*) mRNA, normalized to *EEF1A1* (2^–ΔCT^) in bat SI^ORG^ or alv^ORG^ treated with or without recombinant bat IFNλ3-like (equivalent to 1000 U/ml universal IFNα2) for 8 h. A two-sided Student’s t-test was used to compare IFNL3-like mRNA levels 8 h after treatment to mock-treated controls. ***P<0.001, ****P<0.0001.

Among *R. aegyptiacus* ISGs that piqued our interest was the putative *IFNL3*-like gene (*LOC107521776*), which was induced by IFN in nasal^ORG^, alv^ORG^ or SI^ORG^, particularly after 8 h of stimulation with bat IFNλ1-like compared to mock-treated control cultures (Fig. 7a). RT–qPCR analysis confirmed the bulk RNA-seq data and indicated that *IFNL3*-like was significantly upregulated at 4 and 8 h after treatment with IFNλ1-like in bat SI^ORG^ or bat alv^ORG^, but not in human SI^ORG^, despite an equally strong induction of the ISG *MX1* (Fig. 7b). Treatment of bat SI^ORG^ with home-made recombinant bat IFNλ3-like (Methods) for 8 h induced the expression of ISGs (for example, *BST2*, *IFIT1*, *DDX60*, *IFI6*, *IRF7*, *OAS3*, *ZNFX1*) to a similar extent as universal-IFNα2-treated SI^ORG^ (Fig. 7c and Extended Data Fig. 9a,b), confirming its function as IFN. Notably, recombinant IFNλ3-like, derived from the second annotated *IFNL3*-like gene in *R. aegyptiacus* (*LOC107520938*), also triggered ISG expression after 8 h of treatment in SI^ORG^ compared to mock-treated controls (Extended Data Fig. 9c,d).

To determine whether *IFNL3*-like (*LOC107521776*) was a canonical ISG, we performed bulk CRISPR–Cas9 editing of *IRF9*, the key transcription factor that mediates signaling by both type I and type III IFNs^36^. *IRF9* gene perturbation in bat SI^ORG^ resulted in an almost complete loss of the IFNλ1-like-dependent (Fig. 7d), but not VSV-eGFP-dependent (Fig. 7e), induction of *IFNL3*-like. By contrast, *MX1* gene expression in bat SI^ORG^ was largely abolished after *IRF9* perturbation, both in response to IFNλ1-like and VSV infection (Fig. 7d,e), indicating both virus-dependent and virus-independent pathways for the induction of *IFNL3*-like. Additionally, we observed that recombinant IFNλ3-like protein robustly induced its own expression in bat SI^ORG^ and alv^ORG^ (Extended Data Fig. 9e), indicating that IFNλ3-like might control a positive autoregulatory feedback loop. In summary, we characterized the functional capacity of three type III IFNs to induce antiviral gene responses in *R. aegyptiacus* epithelial organoids and identified a unique role for *IFNL*3-like (*LOC107521776*), which can also function as an ISG itself.

### Type I and type III IFNs protect bat organoids from viral infection

**Fig. 8:**
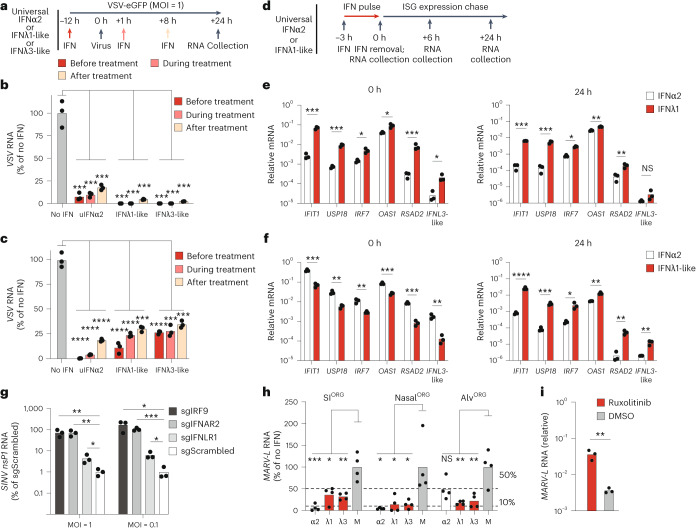
Type I and type III IFNs protect bat organoids from zoonotic virus infection. **a**, Experimental workflow showing the administration of universal IFNα2, IFNλ1-like or IFNλ3-like 12 h before, during or 8 h after treatment with VSV infection of bat nasal^ORG^ or SI^ORG^. Intracellular VSV viral RNA was measured 24 h after infection. **b**,**c**, RT–qPCR analysis of intracellular *VSV-NP* viral RNA in bat SI^ORG^ (*n* = 3) (**b**) or nasal^ORG^ (*n* = 3) (**c**), normalized to *EEF1A1* (2^−Δ*C*t^) and expressed as a percentage relative to no IFN control. A two-sided Student’s *t*-test was used to compare *VSV* viral RNA levels across before, during and after treatment, and no IFN conditions, comparing each IFN treatment to no IFN control. **d**, Experimental workflow showing bat nasal^ORG^ or SI^ORG^ treated with universal IFNα2 or bat IFNλ1-like for 3 h, followed by washout and incubation in IFN-free medium for 24 h and RNA collection at 0, 3 and 24 h after IFN washout. **e**,**f**, RT–qPCR analysis of ISGs (normalized to *EEF1A1*) in bat SI^ORG^ (*n* = 3) (**e**) or nasal^ORG^ (*n* = 3) (**f**) treated with universal IFNα2 or bat IFNλ1-like at 3 h (left) or 24 h (right) after IFN removal. A two-sided Student’s *t*-test was used to compare ISG mRNA levels between treatments. **g**, RT–qPCR analysis of intracellular SINV-eGFP^nsP2-P726G^ *nsP1* RNA (normalized to *EEF1A1* (2^−Δ*C*t^) and expressed as fold change relative to the mean of the sgScrambled control) in bat SI^ORG^ engineered with Cas9 and targeted guide RNA (sgIRF9, sgIFNAR2, sgIFNLR1) or control (sgScrambled), infected with SINV-eGFP^nsP2-P726G^. A two-sided Student’s *t*-test was used to compare *SINV nsP*1 viral RNA levels between treatment groups. nsP1, nonstructural protein 1. **h**, RT–qPCR analysis of intracellular *MARV-L* viral RNA (normalized to EEF1A1) in bat SI^ORG^, nasal^ORG^ or alv^ORG^ (*n* = 4) treated with or without IFNs during a 3-day MARV infection. A two-sided Student’s *t*-test was used to compare *MARV* viral RNA levels between IFN-treated and untreated controls. **i**, RT–qPCR analysis of *MARV-L* viral RNA in bat alv^ORG^ treated with or without 5 µM ruxolitinib during a 3-day infection. A two-sided Student’s *t*-test was used to compare *MARV* viral RNA levels between ruxolitinib and dimethylsulfoxide (DMSO) mock-treated alv^ORG^ and *MARV*-infected alv^ORG^. *P < 0.05, **P < 0.01, ***P < 0.001, ****P < 0.0001.

To assess the antiviral potential and kinetic differences of type I and type III IFN responses in bat organoids, we infected bat nasal^ORG^ or SI^ORG^ with VSV-eGFP, a fast-replicating virus sensitive to bat IFN^4^, and treated them with recombinant universal IFNα2, bat IFNλ1-like or bat IFNλ3-like, 12 h before infection (before treatment), during infection (co-treatment) or 8 h after infection (after treatment) (Fig. 8a). RT–qPCR measurement of intracellular viral RNA indicated that all three IFNs strongly protected both bat nasal^ORG^ and SI^ORG^ from viral infection in all treatment schemes (Fig. 8b,c). IFN treatment before infection reduced intracellular viral RNA to less than 1–10%, whereas treatment after infection reduced it to less than 20–30% of the levels observed in infected, untreated organoids (Fig. 8b,c).

**Extended Data Fig. 10:**
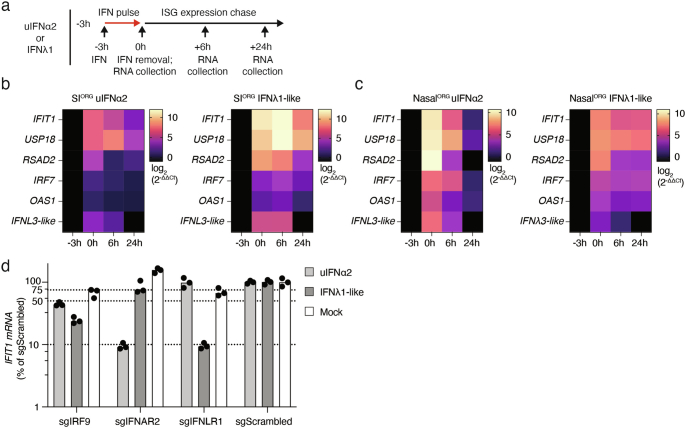
10: Bat type I/III IFN drive antiviral responses with different kinetic profiles in bat SI^ORG^ or Nasal^ORG^. **a**, Experimental workflow to assess the temporal induction and maintenance of ISG expression. Bat Nasal^ORG^ or SI^ORG^ are treated with universal IFNα2 (uIFNα2) or bat IFNλ1-like for 3 hours, followed by washout and incubation in IFN-free medium for 24 hours. **b**, Heatmaps showing RT-qPCR analysis of selected ISGs in bat SI^ORG^ (n = 3), with expression values calculated using the 2 ^-^^ΔΔCT^ method: first normalized to *EEF1A1* (2^–ΔCT^), then to baseline levels at −3 h (before treatment). Timepoints shown include before IFN treatment (−3 h), immediately after treatment (0 h), and various hours post-interferon removal (+6h, +24h). Each heatmap value represents the average normalized ISG expression across three biological replicates. Left, uIFNα2. Right, IFNλ1-like treatment. **c**, same as in **(b)** but for bat nasal^ORG^. **d**, RT-qPCR analysis of *IFIT1* (normalized to *EEF1A1*(2^–ΔCT^), then expressed as a percentage relative to the average sgScrambled control in bat nasal^ORG^ engineered with Cas9 and a targeted guide RNA (sgIRF9, sgIFNAR2 or sgIFNLR1) or control guide RNA (sgScrambled) (n = 3 each), treated or not for 8 h with uIFNα2 or bat IFNλ1-like.

To examine the difference in antiviral response kinetics between universal IFNα2 and bat IFNλ1-like in bat nasal^ORG^ or SI^ORG^, we measured the induction and persistence of ISG expression (*IFIT1*, *USP18*, *IRF7*, *OAS1*, *RSAD2* and *IFNL3*-like) over a 24-h period using a pulse-chase experiment in which organoids were treated with universal IFNα2 or bat IFNλ1-like for 3 h, followed by washout and incubation in IFN-free culture medium (Fig. 8d). We observed stronger expression of all measured ISGs in bat SI^ORG^ treated with bat IFNλ1-like compared to universal IFNα2, while the reverse was true in bat nasal^ORG^ (Extended Data Fig. 10a–c). Despite this difference, treatment with bat IFNλ1-like resulted in significantly longer-lasting expression of all tested ISGs (except *IFNL3*-like), maintaining increased levels up to 24 h after IFN removal in both organoid types, in contrast to universal IFNα2 treatment, which led to ISG expression returning to basal levels by the 24-h time point (Fig. 6e,f and Extended Data Fig. 10b,c). These data suggested differences in the sensitivity and timing of the response of *R. aegyptiacus* intestinal and respiratory ECs to type I and type III IFNs.

To further assess the requirement for endogenous IFN signaling in cell-intrinsic viral restriction, we used bulk CRISPR–Cas9 editing to target *IRF9*, the type I IFN receptor *IFNAR2* and the type III IFN receptor *IFNLR1* in bat nasal^ORG^ (knockout (KO) organoids). Gene editing significantly reduced the sensitivity of knockout organoids to exogenous IFNs, but did not completely abolish signaling, a limitation inherent in bulk CRISPR–Cas9 editing (Extended Data Fig. 10d). *IRF9*, *IFNAR2* and *IFNLR1* knockout organoids challenged with SINV-eGFP-nsP2-P726G, an attenuated variant of the alphavirus Sindbis virus (SINV) carrying a point mutation in the *nsP2* gene and eGFP^38^, accumulated significantly more intracellular viral RNA compared to scrambled guide control nasal^ORG^ (Fig. 8g), suggesting that both type I and III IFN receptor signaling were important for viral restriction.

Lastly, we evaluated the ability of type I and type III IFNs to restrict MARV replication in bat ECs. Treatment with universal IFNα2, bat IFNλ1-like or bat IFNλ3-like at the time of MARV infection significantly reduced intracellular viral RNA in bat nasal^ORG^, alv^ORG^ or SI^ORG^ compared to mock-treated organoids (Fig. 8h). Moreover, treating MARV-infected bat alv^ORG^ with the JAK–STAT signaling inhibitor ruxolitinib during infection significantly increased intracellular viral RNA (Fig. 8i), indicating that endogenous IFN signaling was required to limit MARV replication. In conclusion, our findings identified distinct antiviral roles for type I and type III IFNs in *R. aegyptiacus* ECs, supporting both rapid induction and prolonged antiviral protection.

## Discussion

In this study, we developed a sustainable organoid platform to model epithelial innate immunity and enable functional genetic studies in *R. aegyptiacus*. Our findings demonstrate that bat adult stem cells retain their full differentiation potential in vitro, faithfully recapitulating the cellular diversity and gene expression profiles found in the native epithelium. Cross-species comparisons with human organoids revealed an enhanced antiviral gene expression signature in bat organoids both at baseline and after infection. By delineating type I and type III IFN responses, we identified their critical roles in restricting viral infection through both rapid induction and sustained antiviral activity.

Based on our findings, we propose a model for epithelial antiviral immune responses in *R. aegyptiacus* in which basal expression of ISGs, partially driven by type I IFNε, provides a constitutive level of defense and primes ECs against infection, with complement gene expression potentially contributing to this baseline protection. Upon viral sensing, the induction of type I IFNβ, type III IFNλ1-like and IFNλ3-like confers robust antiviral protection. Notably, the consistent and strong induction of type III IFNs, irrespective of viral species, coupled with their long-lasting protective effects and unique virus-independent regulation of IFNλ3-like, underscores the pivotal role of IFNλ in limiting viral replication at epithelial surfaces in *R. aegyptiacus*.

Therefore, asymptomatic MARV infection in *R. aegyptiacus*^7,35^ could result from the strong induction of innate antiviral immunity at infected mucosal barrier tissues, which would restrict virus dissemination and concurrently limit excessive inflammation. Supporting this hypothesis, pharmacological inhibition of innate immune responses using dexamethasone enhances MARV replication in *R. aegyptiacus*, causing MARV-like disease in infected animals^39^. On the other hand, an inability of humans and nonhuman primates to mount a potent early antiviral response at mucosal surfaces against MARV infection may permit systemic virus dissemination, leading to the observed uncontrolled virus replication, widespread inflammation and lethal disease^40^. The broad cellular distribution of the major filovirus host entry factor NPC1 probably contributes to the detrimental disease outcome when local virus restriction is impaired. A mechanistic explanation for the observed species-specific differences in antiviral responses may lie in the ability of bats to overcome innate immune antagonists encoded by filoviruses^41^. Two such viral proteins, VP35, a dsRNA-binding protein and RIG-I antagonist, and VP40, which inhibits JAK–STAT signaling, may function differently in *R. aegyptiacus* ECs compared to human cells or may fail to block type I and type III IFN signaling altogether^42,43^. The elevated basal expression of ISGs observed in this study and shown previously^1,33,44^, including pattern recognition receptors and downstream signaling proteins, may further lower the intracellular threshold for viral sensing in *R. aegyptiacus* ECs, enhancing their capacity to detect and respond to viral infection. While most of our experiments focused on the antiviral effects of IFN against VSV, we also observed that type III IFNλ1-like and IFNλ3-like effectively inhibited MARV replication in bat organoids. This finding suggests that IFNλ could be explored as a therapeutic strategy to prevent lethal filovirus infections in humans and nonhuman primates.

This study has limitations. Mucociliary differentiation is a hallmark of the respiratory epithelium; while our bat organoid models successfully formed ciliated and specialized ECs (for example, microfold cells, tuft and brush cells, and EECs), goblet cells were rare unless stimulated with IL-13. Similar to the absence of Paneth cells in human SI organoids^45^, we speculate that in vivo microbial cues may trigger cytokine production that is absent or not supported by the commercial human differentiation media used. To address this, an optimized differentiation cocktail, probably requiring species-specific growth factors and tailored culture conditions, needs to be developed. Additionally, in vitro models require careful interpretation when extrapolating findings to in vivo contexts, particularly given the absence of classical immune cells, which have a crucial role in systemic antiviral immunity. Incorporating immune cells into organoid cultures could provide valuable insights into epithelial immune crosstalk during viral infections. Moreover, direct exposure of epithelial organoids may not fully replicate the natural infection dynamics at epithelial surfaces in vivo. Future studies should validate the role of epithelial immune responses in viral clearance through mucosal inoculation rather than systemic injection, potentially also combining these experiments with pharmacological inhibition or exogenous stimulation of the bat mucosa with type I or type III IFN to further explore their roles during infection in Egyptian fruit bats.

Our bat organoid platform provides a valuable tool for investigating genetic pathways and complements the limited in vivo studies possible in bats. Despite its limitations, this organoid-based approach bridges in silico^4^, in vitro^46^ and in vivo^7^ research, advancing our understanding of bat antiviral immunity and how bats can harbor pathogenic zoonotic viruses without disease. It also enables direct comparison with human and animal organoids, providing insight into cross-species viral dynamics and epithelial responses to infection. Together, these advances provide a foundation for future mechanistic studies using complex, non-immortalized in vitro models of *R. aegyptiacus*.

## Methods

### Bat tissue collection and human primary cell isolation

*R. aegyptiacus* bat tissue for organoid generation was derived from a breeding colony at the Friedrich-Loeffler-Institut in Germany. Collection was carried out in accordance with European and national animal welfare regulations applicable in the federal state of Mecklenburg-Western Pomerania, Germany. Freshly collected organ tissue from male and female bats was washed in PBS and cut into 3–5-mm pieces before placing up to four individual pieces into a 1-ml cryostorage tube containing 0.5 ml of CryoStor CS10 freezing medium (STEMCELL Technologies). Vials were immediately transferred to a cryostorage container (Mr. Frosty) and frozen overnight at −70 °C, followed by liquid nitrogen storage the following day. In total, nasal respiratory, lung and small intestinal tissue from three individual bats (males, *n* = 2; females, *n* = 1) and tracheal tissue from two individual bats (males, *n* = 2) were obtained and included in this study.

Human nasal ECs were collected from a brush biopsy of the middle turbinate section of a healthy female donor and placed into Advanced DMEM/F-12 (Gibco). After collection of cells via centrifugation, individual cells were obtained using TrypLE Express (Gibco) enzyme dissociation, followed by plating onto PureCol-treated (Advanced BioMatrix, diluted 1:30 in PBS or Advanced DMEM/F-12) cell culture dishes in PneumaCult-Ex Plus Medium (STEMCELL Technologies). Nasal ECs were expanded for up to two additional passages in PneumaCult-Ex Plus Medium and cryopreserved in liquid nitrogen in CryoStor CS10 freezing medium. Experiments were approved by the ethics commission of the Medical University of Vienna (no. ECS 2234/2021). ALI quality-controlled human bronchial ECs were purchased from PromoCell (cat. no. C-12640) and were initially recovered in PromoCell airway expansion medium. Cells were further expanded for up to two additional passages in PromoCell airway expansion medium or PneumaCult-Ex Plus Medium and cryopreserved in CryoStor CS10 freezing medium.

### Establishment and maintenance of bat airway organoids

Frozen tissue pieces were quickly thawed in a water bath before transferring to a gentleMACS dissociation tube (Miltenyi Biotec) containing Advanced DMEM/F-12, supplemented with 1:100 GlutaMAX (Gibco), 10 mM HEPES (Gibco), DNase I (Roche) and 1.25 µg ml^−1^ collagenase from *Hathewaya histolytica* (Collagenase from *Clostridium histolyticum*, Sigma-Aldrich). A homogenous cell suspension was obtained by initial disruption of bulk tissue pieces using program m_lung_01, followed by dissociation using protocol 37C_mLIDK_01 in a gentleMACS dissociator (Miltenyi Biotec). Dissociation was stopped by adding an equal volume of 10% FBS (Heat-inactivated, Gibco, A5670801) in Advanced DMEM/F-12. The cell suspension was strained through a 70-µm filter and cells were collected by centrifugation at 300*g* for 5 min. To remove red blood cells, the cell pellet was resuspended and incubated in 1 ml of red blood cell lysis buffer (Roche) for 5 min. Cells were resuspended in Advanced DMEM/F-12 and counted using Trypan Blue to estimate the live and dead cell number percentages. If cell viability was below 60%, dead cells were removed using annexin V-based magnetic bead selection according to the manufacturer’s instructions (STEMCELL Technologies). Briefly, cells were collected using centrifugation, resuspended in 1 ml PBS, supplemented with 2% FCS and 1 mM CaCl_2_ (Sigma-Aldrich), and transferred to a 5-ml fluorescence-activated cell sorting (FACS) tube. Then, 50 µl of annexin V biotin beads were added and incubated for 5 min; 50 µl of magnetic RapidSpheres were added for 3 min. Then, 1.5 ml of PBS, supplemented with 2% FCS and 1 mM CaCl_2_, was added and the tube was subsequently placed into a magnet. After a 3-min separation, unbound viable cells were decanted, collected using centrifugation (300*g* for 5 min) and resuspended in 1 ml Advanced DMEM/F-12 before counting.

For nasal and tracheal epithelial tissues, cells were either expanded two-dimensionally using the PneumaCult-Ex Plus Medium as described for human airway ECs (see above) or expanded under three-dimensional culture conditions (expanding organoids). To establish expanding basal cell nasal^ORG^, trachea^ORG^ or bronchial^ORG^, 25,000 viable cells were resuspended in 50 µl ice-cold Matrigel (cat. no. 356255, Corning) and plated onto prewarmed 24-well plates. After 15–20 min of Matrigel solidification, basal cell expansion medium (Advanced DMEM/F-12 + 1:100 GlutaMAX + 10 mM HEPES + 1.25 mM *N*-acetyl-l-cysteine (Sigma-Aldrich) + 1:50 B-27 Supplement (Gibco) + 1:100 N-2 Supplement (Gibco) + 50 ng ml^−1^ human EGF (Gibco) + 25 ng ml^−1^ human FGF10 (STEMCELL Technologies) + 25 ng ml^−1^ human Noggin (STEMCELL Technologies) + 10 vol% conditioned medium containing R-spondin 1 (produced from the HA-R-Spondin 1-Fc 293T cell line, Trevigen) + 500 nM A-83-01 (Selleck Chemicals) + 1:500 Primocin (InvivoGen) + 10 µM Y-27632 ROCK inhibitor (STEMCELL Technologies)) was added. (The ROCK inhibitor was used only during the first 2 days.) The medium was changed every 2–3 days. Organoids were split every 7–9 days by collecting them in 1 ml cold Advanced DMEM/F-12 and quick centrifugation (short acceleration to 5,000*g*, ~3–4 s), followed by removal of the supernatant containing dead cells and residual Matrigel and resuspension in 500 µl of TrypLE Express enzyme. After single-cell dissociation at 37 °C for 5 min, 500–1,000 µl of Advanced DMEM/F-12 was added and cells were collected by centrifugation at 300*g* for 4 min, followed by cell resuspension in 1,000 µl Advanced DMEM/F-12 and centrifugation at 300*g* for 4 min. Five thousand viable single cells were reseeded in 50 µl Matrigel droplets in 24-well plates. Expanding organoids with fewer than ten passages were used for the differentiation experiments or cryopreserved in CryoStor CS10 freezing medium. For differentiation at the ALI, 100,000 nasal^ORG^ or bronchial^ORG^ derived ECs were seeded in 200 µl basal cell expansion medium or PneumaCult-Ex Plus Medium supplemented with 10 µM Y-27632 onto PureCol-treated Transwell inserts (0.4 µm pore size, 24-well plate, Corning). The apical and basal chamber medium was replaced every 2–3 days until cells reached confluency, after which they were air-lifted by removing medium in the apical chamber and replacing the basal chamber medium with complete PneumaCult ALI medium (STEMCELL Technologies) + 1:500 Plasmocin. Cultures were differentiated at the ALI for 30–40 days with medium changes every 3–4 days and weekly apical PBS washes to remove mucus or dead cells. Alternatively, organoids were differentiated three-dimensionally by replacing the expansion medium with PneumaCult ALI medium supplemented with 10 µM DAPT (Sigma-Aldrich) at day 4 or 5 after initial expansion, for 15–20 days. To prevent loss of differentiating three-dimensional organoids because of attachment to the plastic dish, organoids were collected in Cell Recovery Solution (Corning) and left on ice for 30 min to remove Matrigel. Organoids were then reseeded into fresh Matrigel and overlayed with PneumaCult ALI medium supplemented with 10 µM DAPT. This step was necessary after a total incubation period of ~10 days.

To determine the optimal expansion medium for lung organoids containing bronchial or bronchiolar basal and alveolar AT2 progenitor cells, 25,000 dissociated viable lung cells were seeded per 50 µl Matrigel droplet, followed by the addition of lung basal medium (Advanced DMEM/F-12 with 1:100 GlutaMAX, 10 mM HEPES, 1.25 mM *N*-acetyl-l-cysteine, 1:50 B-27 Supplement, 1:100 N-2 Supplement, 50 ng ml^−1^ human EGF, 1:500 Primocin and 0.002% heparin (Sigma-Aldrich)) supplemented with 10 µM Y-27632 (until the first medium change after 3 days) and additional growth factors (shown in Extended Data Fig. 1c). These factors included 50 vol% conditioned medium containing human WNT3A (the l-Wnt3a cell line was a gift from H. Clevers), 10 vol% conditioned medium containing R-spondin 1, human FGF10 (25 ng ml^−1^), human Noggin (25 ng ml^−1^), CHIR9902 (3 µM, STEMCELL Technologies), SB431542 (10 µM, STEMCELL Technologies) and 1 µM BIRB796 (1 µM, STEMCELL Technologies). Lung bronchial^ORG^ were cultured in the basal cell expansion medium described for nasal^ORG^ and trachea^ORG^. To generate bat alveolar organoids (alv^ORG^), mixed lung progenitor organoids (lung^MIX-ORG^) were established from 25,000 dissociated viable lung cells grown in 50 µl Matrigel droplets and alveolar expansion medium (lung basal medium supplemented with 25 ng ml^−1^ human FGF10 + 25 ng ml^−1^ human Noggin + 3 µM CHIR9902 + 10 µM SB-431542 (STEMCELL Technologies) + 1 µM BIRB796 + 10 µM Y-27632 for the first 3 days after passaging) and expanded at a ratio of 1:3 as described above for nasal^ORG^, trachea^ORG^ or bronchial^ORG^ for one to three passages. AT2 and non-AT2 progenitor cells (bronchiolar basal cells) were separated using FACS with LysoTracker Red DND-99 dye (Thermo Fisher Scientific) for specific staining of alveolar AT2 cells. To accomplish this, LysoTracker dye (1:10,000 dilution) was added to lung^MIX-ORG^ cultures for 2 h, followed by single-cell dissociation using TrypLE Express enzyme at 37 °C and sorting of LysoTracker^+^ (AT2 cells for alv^ORG^) and LysoTracker^−^ (basal cells for bronchial^ORG^) lung epithelial progenitor cells. Sorted cells were placed in basal cell expansion medium (LysoTracker^−^ cells, bronchial^ORG^) or complete alveolar expansion medium (LysoTracker^+^, alv^ORG^), supplemented with 10 µM Y-27632 for the first 3 days. Lung bronchial^ORG^ were split using single-cell dissociation as described for nasal^ORG^ and trachea^ORG^ Alv^ORG^ were passaged every 10–14 days by collecting them in cold Advanced DMEM/F-12, followed by resuspension in TrypLE Express and fragment dissociation for 3–5 min at room temperature. Fragments were collected in Advanced DMEM/F-12 and passaged at a split ratio of 1:4–1:8 and reseeded in 50 µl Matrigel droplets into new 24-well cell culture plates, followed by the addition of complete alveolar expansion medium.

### Establishment and maintenance of bat SI organoids

Bat SI cells were obtained from frozen tissue as described above for airway cells. To establish SI^ORG^, 25,000 cells were seeded in 50 µl Matrigel and overlayed with SI organoid expansion medium containing Advanced DMEM/F-12, 10 vol% conditioned medium containing R-spondin 1, 50 vol% conditioned medium containing human WNT3A (the l-Wnt3a cell line was a gift from H. Clevers), 1:100 GlutaMAX, 10 mM HEPES, 1 mM *N*-acetyl-l-cysteine, 1:50 B-27 Supplement, 50 ng ml^−1^ human EGF, 100 ng ml^−1^ human Noggin, 100 ng ml^−1^ human IGF1 (STEMCELL Technologies), 50 ng ml^−1^ human FGF2 (STEMCELL Technologies), 500 nM A 83-01, 1:500 Primocin and 10 µM Y-27632 (only for the first 2 days). A full medium change was done after 2–3 days. Passaging was done by collecting SI^ORG^ grown in Matrigel droplets in cold Advanced DMEM/F-12 and quick centrifugation (short acceleration to 5,000*g*, ~3–4 s). The supernatant containing dead cells and Matrigel was removed and the cell pellet resuspended in 500 µl of TrypLE Express. After 3–5-min TrypLE Express assisted fragmentation of SI^ORG^ at room temperature, 500 µl of cold Advanced DMEM/F-12 was added to the suspension and organoids were fragmented using manual pipetting (five to ten times up and down pipetting using a P1000 pipette). After quick centrifugal collection (short acceleration to 5,000g, ~3–4 s) of organoid fragments, the supernatant was removed and 1,000 µl Advanced DMEM/F-12 was added. To obtain the correct split ratio, an aliquot of the organoid fragment suspension was transferred to a new tube and cells were collected using quick centrifugation (short acceleration to 5,000g, ~3–4 s). Finally, organoid fragments were seeded in ice-cold Matrigel. We typically split SI^ORG^ once a week at a 1:6–1:12 split ratio.

Organoid differentiation was initiated by replacing the culture medium 3 days after passaging with differentiation medium containing Advanced DMEM/F-12, 10 vol% conditioned medium containing R-spondin 1, 1:100 GlutaMAX, 10 mM HEPES, 1 mM *N*-acetyl-l-cysteine, 1:50 B-27 Supplement, 50 ng ml^−1^ human EGF, 100 ng ml^−1^ human IGF1, 50 ng ml^−1^ human FGF2, 500 nM A 83-01 and 1:500 Primocin. SI^ORG^ differentiation was carried out for another 2–4 days before using the organoids for the experiments. Human SI^ORG^ were established from healthy duodenal biopsy specimens by isolating intestinal crypts (full ethical approval: REC-12/EE/0482). We received a vial of frozen organoids^47^ and cultured them alongside bat organoids in expansion or differentiation medium as detailed above.

### Production of Egyptian fruit bat IFNλ

The coding sequences lacking the signal peptide of *R. aegyptiacus* *IFNL1*-like (*LOC107521777*) and *IFNL3*-like (*LOC107521776* or *LOC107520938*) were obtained using RT–qPCR from RNA of poly(I:C)-stimulated (InvivoGen) bat nasal ECs. Stimulation was carried out by transfecting 30,000 cells with 100 ng of high-molecular-weight poly(I:C) in 10 µl Opti-MEM I (Gibco) + 0.15 µl Lipofectamine 3000 (Thermo Fisher Scientific) for 8 h before collecting RNA. The PCR fragments were cloned into a PiggyBac mammalian expression vector (cat. no. PB210PA-1, System Biosciences), modified to harbor a Secrecon-AA signal peptide^48^ for secretion at the N terminus and 6× His-tag at the C terminus. The expression vector was further modified to contain an internal ribosome entry site-puromycin selection cassette. A stable IFNλ-expressing cell line was obtained by transfecting one million Lenti-X cells (293T, Takara Bio) with 500 ng Super PiggyBac Transposase vector and 1,250 ng of PiggyBac Transposase mammalian expression vector using Lipofectamine 3000 according to the manufacturer’s instructions. Stable cells were selected with 1 µg ml^−1^ Puromycin and expanded for 2 weeks. For the collection of bat IFNλ, cells were seeded at high density (1.2 million cells per one well of a six-well plate) and incubated for 3 days. The supernatant was cleared using centrifugation, filtered through a 0.22-µm syringe filter and stored in aliquots at −70 °C. Biological activity was determined by adding diluted amounts of bat IFNλ supernatant to primary bat nasal ECs and measuring the induction of ISGs (*IFIT1*, *OAS1*, *RTP4*) using RT–qPCR, 8 h after stimulation. Universal IFNα2 (cat. no. 11200-1, PBL Assay Science) at 1,000 U ml^−1^ was used as the positive control; unmodified Lenti-X culture supernatant and ruxolitinib (1 µM, InvivoGen) inhibition were used as negative controls. For the stimulation experiments with bat IFNλ, we used bat IFNλ1-like or bat IFNλ3-like containing supernatants at amounts needed to reach *IFIT1* mRNA induction, equivalent to induction with universal IFNα2 (~1:50–1:100 IFNλ dilution equivalent to 1,000 U ml^−1^ universal IFNα2).

### IFN stimulation assays

Bat nasal^ORG^, alv^ORG^ or SI^ORG^, and human SI^ORG^, were grown in expansion medium, collected in cold Advanced DMEM/F-12 using quick centrifugation (short acceleration to 5,000g, ~3–4 s), which was followed by resuspension of the organoid pellet in 500 µl TrypLE Express and incubation for 2 min at room temperature; this was enough to remove excess Matrigel and produce large organoid fragments. Equivalent amounts of Advanced DMEM/F-12 were then added and organoid fragments collected using quick centrifugation. The pellet containing the organoid fragments was resuspended in organoid expansion medium (bat alv^ORG^ without the mitogen-activated protein kinase inhibitor BIRB796) and seeded onto Matrigel-coated wells of a 96-well plate (1:50 dilution of Matrigel in cold Advanced DMEM/F-12 for 1 h at 37 °C). One 50-µl Matrigel droplet (one well of a 24-well plate) of a 7–10-day-old (3–4 days for SI^ORG^) organoid culture was used per six wells of a 96-well plate and resuspended at 100 µl of medium per condition (600 µl of medium per Matrigel droplet containing organoids, approximately 300,000 cells or 50,000 cells per well in a 96-well plate). After 1–2 days after seeding, diluted amounts of recombinant bat IFNλ (dose equivalent to 1,000 U ml^−1^ of universal IFNα2), 1,000 U ml^−1^ of universal IFNα2 or 100 ng ml^−1^ of recombinant human IFNλ1 (PeproTech) were added for 8 h unless otherwise indicated. RNA was collected by removing culture medium and adding 200 µl of KingFisher RNA lysis buffer (Thermo Fisher Scientific, supplemented with 1 M dithiothreitol (DTT) (Roche)). Lysates were stored at −70 °C for up to 2 weeks before RNA extraction. To stimulate human bronchial^ALI^, recombinant IFN was diluted in Gibco OptiPRO serum-free medium (100 ng ml^−1^ human IFNλ1 or 1,000 U ml^−1^ universal IFNα2) and added to the apical chamber of the culture insert for the indicated amount of time (8 h unless otherwise indicated). RNA was collected by removal of culture medium from the apical and basal chamber, followed by the addition of 400 µl DTT-supplemented KingFisher RNA lysis buffer and subsequently transferred to collection tubes for storage at −70 °C.

### Viruses and viral infection

All experiments involving MERS-CoV and MARV infection were done in compliance with the Swedish Public Health Agency guidelines in the appropriate biosafety level 3 (MERS-CoV) and biosafety level 4 (MARV) laboratories. All experiments involving SeV (murine respirovirus), IAV subtype H1N1 or VSV-eGFP infections were performed in a biosafety level 2 laboratory in compliance with the health and biosafety committee of the Vienna BioCenter. Virus strains used were MARV Musoke strain (GenBank accession DQ217792), MERS-CoV (EMC/2012), IAV H1N1 (A/WS/33, VR-1520, ATCC), SeV (cat. no. NR-3227, BEI Resources) and VSV-eGFP (Indiana strain modified to express eGFP, gift from M. Hein). SINV encoding eGFP and harboring a mutation in the *nsP2* gene (P726G) was constructed using PCR site-directed mutagenesis of the pBG218 rescue plasmid (Kerafast) encoding a replication-competent SINV genome. Virus rescue was carried out by transfecting 70% confluent BHK-21 cells (ATCC) with the pBG218-nsP2-P726G plasmid using Lipofectamine 3000 (120 ng per 1 × 10^6^ cells) in standard D10A cell culture medium (DMEM + 10% FCS + 1× GlutaMAX + penicillin-streptomycin (100 U ml^−1^)). Four hours after transfection, the medium was changed to D5A (as D10A but with 5% FCS) and cells were incubated for 48 h before the supernatant was collected and titrated on Vero E6 cells using the 50% tissue culture infectious dose assay. Virus stocks were generated by one more round of passaging on Vero E6 (MOI = 0.1) cells before using for the infection experiments (passage two used for the experiments).

For the infection of bat nasal^ORG^, alv^ORG^ or SI^ORG^, organoid fragments were seeded on Matrigel-coated 96-well plates as described above for IFN stimulation for 2 days before infection. For infection, the medium was removed and replaced with absorption medium (Gibco OptiPRO serum-free medium, 1:100 GlutaMAX, 10 mM HEPES) containing virus at various doses (estimated MOI range = 0.1–1). After 2–5 h of absorption (1 h for SeV, SINV, H1N1 and VSV-eGFP), the inoculum was removed and replaced with fresh organoid growth medium (that is, differentiation medium for SI organoids or alveolar growth medium without BIRB796 mitogen-activated protein kinase inhibitor for alv^ORG^). The culture medium was further supplemented with IFNs or inhibitors for the specific experimental variations used in the study (for example, IFN protection assays or JAK inhibition using ruxolitinib). RNA was collected at the indicated time points by removing the supernatant and adding TRIzol. TRIzol lysates were transferred into separate tubes and handled according to the respective biosafety protocols before storage at −70 °C and RNA extraction.

For 24-well Transwell-grown ALI cultures, the apical side was first washed once with PBS to remove excess mucus and dead cells. Infection was initiated by adding 100 µl of virus inoculum containing 100,000 PFU (MOI range estimate = 0.5–1) in virus absorption medium (OptiPRO serum-free medium, 1:100 GlutaMAX, 10 mM HEPES) to the apical chamber of the ALI cultures. After incubation for 5 h in a tissue culture incubator, the inoculum was removed and fresh ALI medium was added to the basal chamber. At the indicated time points after infection, the medium was removed and RNA was collected by adding TRIzol, and transferred to separate collection tubes for storage at −70 °C and RNA extraction.

### CRISPR–Cas9 gene editing in bat organoids

Oligonucleotides containing guide RNA spacer sequences with overhangs were ordered (‘CAAC’ for the top oligonucleotides, ‘AAAC’ for the bottom oligonucleotides) and cloned into the lentiCRISPRv2 using Golden Gate cloning. In brief, top and bottom strand oligonucleotides (1 µl of 100 µM each) were phosphorylated with T4 polynucleotide kinase (New England Biolabs) for 30 min at 37 °C, denatured for 5 min at 95 °C and slowly cooled to 25 °C (0.1 °C per second ramp rate) for annealing. One microliter of 1:100 diluted product was used for Golden Gate cloning into lentiCRISPRv2 puro plasmid (plasmid no. 52961, Addgene) using 11 cycles of 5 min T4 ligase ligation at 16 °C and 5 min of BsmbI digestion at 37 °C, followed by a final digestion of 15 min at 37 °C. Two microliters of the reaction was transformed into Stbl3 *Escherichia Coli* cells and grown at 34 °C. After plasmid isolation, lentivirus was produced by transfecting 95% confluent Lenti-X cells grown in the wells of six-well plates with 1,000 ng psPAX2 (plasmid no. 12260, Addgene) packaging plasmid, 500 ng of pCMV-VSV-G (plasmid no. 8454, Addgene) and 1,000 ng of cloned lentiCRISPRv2 puro plasmid using Lipofectamine (5 µl P3000, 7 µl L3000). After 6 h, the medium was changed to virus production medium containing Opti-MEM I + 5% FCS + 1:100 GlutaMAX + 1:100 MEM-nonessential amino acids (Gibco) + 1:100 sodium pyruvate + 1:100 penicillin-streptomycin solution. Virus was collected 24 and 48 h after transfection, filtered through a 0.22-µm syringe filter and stored in aliquots at −70 °C. For editing, organoid fragments were prepared as described above for passaging, resuspended in 50 µl of undiluted lentivirus supernatant + 50 µl expansion medium + 5 µM cyclosporin A (Sigma-Aldrich) + 5 µg ml^−1^ Polybrene (Sigma-Aldrich) and spun at 800*g* for 1.5 h at 32 °C in a microcentrifuge. The supernatant was removed and organoid fragments were resuspended in 50 µl of cold Matrigel and seeded as droplets into 24-well plates. Expansion medium containing 5 µM cyclosporin A + 10 µM Y-27632 was added for 24 h, then replaced with standard expansion medium. Puromycin selection (1 µg ml^−1^) was started 60 h after transfection. Organoids were passaged two more times in puromycin selection medium before they were used in the experiments. Loss of function was determined by ISG induction using RT–qPCR (*IRF9*, *IFNLR1* and *IFNAR2*) in response to IFN stimulation. Targeting of *IFNE* was confirmed using PCR amplification of the *IFNE* gene and Sanger sequencing of the amplicon.

### RNA extraction

For RNA extraction involving noninfected material, a semiautomated KingFisher magnetic bead-assisted protocol was used, including DNase I (RNase-free, New England Biolabs) digestion to remove genomic DNA. For virus-inactivated samples in TRIzol, a magnetic bead-assisted semiautomated purification workflow was performed using Direct-zol-96 RNA Kits (Zymo Research), including DNase I digestion to remove genomic DNA. RNA was eluted in 50 µl nuclease-free water and stored at −70 °C for further use.

### RT–qPCR and analysis

RT–qPCR on total RNA (100–500 ng) was performed with random hexamer/oligo(dT) primers containing LunaScript RT SuperMix (New England Biolabs) according to the manufacturer’s instructions. First-strand complementary DNA was diluted 1:6 in nuclease-free water. Two microliters were subsequently used in real-time qPCR reactions containing SYBR Green-based Luna universal dye qPCR mix (New England Biolabs) and gene-specific PCR primers, following the fast cycling protocol outlined in the manufacturer’s instructions. RT–qPCR primers were designed using the Integrated DNA Technologies PrimeQuest tool, using the NCBI transcript ID of interest and qPCR primer and intercalating dye as input options. For the data analysis, Δ*C*_t_ values were first calculated by subtracting the *C*_t_ value measured for the reference gene *EEF1A1* from the *C*_t_ value of a given gene measured from the same sample cDNA (for example, Δ*C*_t_ for gene 1 in sample 1 was defined by calculating *C*_t_ gene 1 (sample 1) minus *C*_t_ *EEF1A1* (sample 1)). Normalized relative expression values were then calculated by raising the negative Δ*C*_t_ value of each sample to the power of two (2^−ΔCt^). This represents the relative expression value of a given gene in each sample in non-logarithmic space. Alternative normalization methods are described in the respective figure legends. Normalized relative expression values were used for visualization and statistical analysis in Prism (GraphPad Software). Primer pairs can be found in Supplementary Table 7.

### Immunofluorescence staining

Organoids grown in Matrigel were collected in Corning Cell Recovery Solution and subsequently fixed in 4% paraformaldehyde (PFA) (Sigma-Aldrich) in PBS for 45 min to 2 h at room temperature, followed by three washes with PBS and storage in PBS at 2–8 °C. Organs were fixed by placing 3–5-mm tissue pieces in 4% PFA solution in PBS for 1 h at room temperature before transferring them to 2–8 °C for overnight fixation. Transwell insert membranes from ALI cultures were washed in PBS and fixed in 4% PFA for 45 min to 2 h at room temperature, followed by three washes with PBS and storage in PBS at 2–8 °C. Next, fixed samples were embedded in paraffin and sectioned at 2-µm thickness. Sample sections were dewaxed before a 30-min citrate buffer (10 mM sodium citrate, pH 6, 0.05% Tween-20) antigen removal step in a water simmer. After allowing sections to cool to room temperature, samples were blocked in PBS buffer containing 5% Donkey Serum (Sigma-Aldrich) (or 2% BSA) and 0.25% Triton X-100 (Sigma-Aldrich) for 30 min. Primary antibody dilutions in PBS containing 3% Donkey Serum (or 1% BSA) and 0.05% Triton X-100 were then added to the samples overnight at 2–8 °C in a humidified chamber. The following day, sections were washed three times in PBS before adding secondary Abs diluted in PBS containing 3% Donkey Serum (or 1% BSA) and 0.05% Triton X-100 or 2 h at room temperature in a humidified dark chamber. Sections were washed three times with PBS, DNA was counterstained with DAPI, and specimens were mounted in mounting medium. The following antibodies were used: anti-Keratin 5 (rabbit, 1:200 dilution, cat. no. SAB4501651, Sigma-Aldrich); anti- acetylated alpha Tubulin (clone 6-11B-1, 1:500 dilution, Santa Cruz Biotechnology); anti-AVIL (rabbit, 1:200 dilution, cat. no. PA5-90703, Thermo Fisher Scientific), anti-SFTPC (rabbit, 1:200 dilution, cat. no. PA5-71680, Thermo Fisher Scientific); anti-E-Cadherin (mouse, 1:200 dilution, cat. no. 610182, BD Biosciences); and anti-IFN-epsilon (mouse monoclonal, 1:200 dilution, cat. no. MAB9147-100, R&D Systems).

### scRNA-seq library preparation and sequencing

Single-cell suspensions of bat tissue were obtained as detailed above for the generation of organoids. After red blood cell removal, cells were collected using centrifugation and viable cells were enriched using an annexin V magnetic bead dead cell removal step according to the manufacturer’s instructions. Recovered viable cells were pelleted using centrifugation, resuspended in Advanced DMEM/F-12 and counted. The single-cell suspension was submitted to an in-house facility to generate 3′ gene expression libraries using the 10x Genomics Chromium Single Cell 3′ Reagent Kit v4.

Single-cell suspensions of organoids were obtained by dissociating organoids or ALI membranes containing cells in TrypLE Express, supplemented with DNase I, for 15–30 min at 37 °C using manual pipetting at 5-min intervals. Cells were collected using centrifugation at 300*g*; viable cells were enriched using an annexin V magnetic bead dead cell removal step according to the manufacturer’s instructions. Recovered viable cells were pelleted using centrifugation at 300*g*, resuspended in Advanced DMEM/F-12 and counted. Single-cell suspensions from identical organoid formats, but different animals or samples, were pooled and submitted to an in-house facility to generate the 3′ gene expression libraries using the 10x Genomics Chromium Single Cell 3′ Reagent Kit v3. Sample pooling was performed to reduce batch variability and cost. For sequencing, a barnyard approach was chosen, where cells from individual samples were mixed and bioinformatically separated downstream using species (human versus bat) or single-nucleotide polymorphism information (different animals). Libraries were sequenced on Illumina instruments using a 150-bp pair-end sequencing mode aiming at 50,000 reads per cell. Individual FASTQ files for downstream analysis were generated by demultiplexing raw reads using Illumina sample indices.

### scRNA-seq analysis

For *R. aegyptiacus* lung, trachea and SI libraries, sequencing reads from 10x Genomics libraries were processed using Cell Ranger (v.7.1). A custom genome index was created using cellranger mkref with the *R. aegyptiacus* reference genome (assembly mRouAeg1.p). Gene expression count matrices were generated using cellranger count. Next, filtered and de-noised count matrices were produced using CellBender^49^ (cellbender remove-background), with the following parameters: --expected-cells 20,000--total-droplets-included 30,000--fpr 0.01--epochs 150. Doublets were identified using Scrublet^50^ on the filtered gene expression matrix (expected doublet rate = 0.1). Downstream analysis was conducted in R using Seurat^51,52^ (v.4.2.1). Gene expression matrices in .h5 format were imported using Read10X_h5 to generate individual Seurat objects. Doublets identified by Scrublet were removed. Seurat objects were filtered to retain cells with more than 300 and fewer than 40,000 detected genes per cell; data were log-normalized (NormalizeData). Cell cycle scores (G2/M and S phase via CellCycleScoring) were computed and regressed out during scaling (ScaleData). The top 2,000 variable features per sample were identified (FindVariableFeatures), and integration anchors were calculated using FindIntegrationAnchors. Samples were integrated with Seurat canonical correlation analysis (CCA) using IntegrateData, followed by data scaling and principal component analysis (PCA) (RunPCA). The number of principal components for UMAP dimensionality reduction was selected using *ElbowPlot*. Cell clusters were identified using FindNeighbours and FindClusters (resolution = 0.5–1). Marker genes were identified with FindAllMarkers (parameters: min.pct = 0.25, log.fc = 0.25, only.pos = TRUE). Clusters were annotated based on known marker genes^28,53,54^ (clustering results in Supplementary Tables 1 and 4). A subset containing EC types was extracted to obtain ECs only. For bat lung and trachea, the clustering and annotation pipeline was repeated to generate a reference dataset. The SI epithelial dataset (SI^T^) was further integrated together with the bat SI^ORG^ (see below).

For the organoid libraries (bat and human nasal^ALI^, bat and human bronchial^ALI^, bat alv^ORG^, and bat and human SI^ORG^), sequencing reads were processed using Cell Ranger (v.7.1) with a custom reference index built from the *R. aegyptiacus* (mRouAeg1.p) and *Homo sapiens* (GRCh38.90) genomes, generated using cellranger mkref. For the bat alv^ORG^ samples, a custom index containing only the *R. aegyptiacus* (mRouAeg1.p) genome was used. CellBender remove-background was applied with the same parameters as described above for the tissue samples. Souporcell^55^ (souporcell_pipeline.py) was used to cluster cells according to single-nucleotide polymorphisms using aligned BAM files (possorted_genome_bam.bam) with the following parameters: -k X, -t 8,--skip_remap True,--ignore True. For downstream analysis, individual Seurat objects were created per 10x library (for example, human + bat SI^ORG^, human + bat nasal^ALI^, human + bat bronchial^ALI^ or bat alv^ORG^). Meta-data from Cell Ranger and Souporcell were added; multiplets and unassigned cells were excluded. For downstream batch correction and sample integration, Seurat objects of individual donor samples (*n* = 3 for bats) were generated using the Souporcell meta-data and SplitObject; next, lists of individual Seurat objects were made. In total, three independent integration sets were analyzed: bat nasal^ALI^ + bronchial^ALI^ (three donors each, six samples); bat alv^ORG^ (three donors); and bat SI^ORG^ (two donors) + SI^ORG-DIFF^ + SI^T^ (four samples). Each integration dataset was processed as follows: For each sample, cells with more than 500 detected genes were retained, followed by log-normalization (NormalizeData). Cell cycle scores were regressed out during data scaling. The top 2,000 variable genes were selected. Integration anchors were identified using FindIntegrationAnchors and samples were integrated with IntegrateData. PCA was performed and UMAP dimensions were chosen based on ElbowPlot (typically 1–30 principal components). Clusters were identified (FindNeighbours, FindClusters) and marker genes were computed (FindAllMarkers). Cell cluster annotation was performed manually using well-established marker genes for the mammalian airway and intestinal epithelium^28,53,54^ (clustering results in Supplementary Tables 2 and 4).

To refine rare cell types in bat airway organoids, *GP2*⁺ microfold cells were identified in the ciliated and microfold cluster based on nonzero expression of *GP2*. Ionocytes were annotated based on the expression of *FOXI1*, *ASCL3* or *PDE1C* (only in nasal^ALI^; bronchial^ALI^ counterparts lacked expression and were therefore reassigned as suprabasal and secretory cells). Neuroendocrine cells expressing *CHGA*, *CHGB* or *SCG3* were detected in nasal^ALI^ only, while cells from bronchial^ALI^ in that cluster that did not were similarly reassigned to the suprabasal and secretory lineage. In the SI^ORG^ and SI^T^ datasets, EECs identified by *CHGA* and *CHGB* expression were subset and reclustered according to the Seurat’s guided clustering vignette.

Human datasets (nasal^ALI^, bronchial^ALI^ and SI^ORG^) were analyzed separately according to the Seurat’s online guided clustering vignette; clusters were annotated based on established marker genes^27,28,54^ (clustering results in Supplementary Table 3).

To compare gene expression across different bat organoid models, Seurat objects were merged into a single object after count normalization using SCTransform. For cross-species comparisons between human and bat organoids, a list of 1:1 orthologs was generated; merged Seurat objects were filtered to retain orthologs only. This involved extracting raw counts using GetAssayData, filtering for orthologs, and reconstructing the Seurat object with the filtered count matrix and associated meta-data. Expression data were then normalized using SCTransform and cell identities were assigned based on species. Differentially expressed genes (DEGs) were identified using FindMarkers (ident.1 = bat, ident.2 = human, min.pct = 0.25, log.fc = 0.25). Plots were generated in Prism (v.9). ISG scores were calculated using Seurat’s AddModuleScore function, with a published list of conserved mammalian ISGs^33^ as input.

One external scRNA-seq dataset was used: small intestinal 10x tissue single-cell sequencing data were downloaded from the Gene Expression Omnibus (accession GSE185224)^30^. ECs from the ileum, duodenum and jejunum of three human donors each were included.

### Pooled 3′-end bulk RNA-seq library preparation

Libraries for 3′-end RNA-seq were generated according to the pooled library amplification for transcriptome expression sequencing protocol (PLATEseq) with modifications^56^. Varying amounts of total RNA were mixed with 1 µl of 10 µM unique molecular identifier (UMI)-containing barcoded anchored oligo(dT) primers (5′ Adapter-BC(8)-UMI(12)-dT(35)VN-3′, consisting of a 5′ adapter sequence, an 8-nucleotide barcode (BC) for sample indexing, a 12-nucleotide unique molecular identifier (UMI) for molecule counting, followed by a 35-mer oligo(dT) stretch to anneal to the poly(A) tail, and a VN anchor to reduce non-specific priming; Supplementary Table 7), 250 nM deoxynucleoside triphosphate mix and heated at 72 °C for 3 min. A reverse transcription mix containing 1x first-strand buffer, 10 mM DTT, 0.15 µl murine RNase inhibitor (New England Biolabs) and 0.2 µl SuperScript III Reverse Transcriptase (Thermo Fisher Scientific) was added to get a total volume of 20 µl. For 48 samples, 20 ng of RNA were used for the reverse transcription reaction and scaled accordingly. Reverse transcription was performed at 50 °C for 30 min, followed by heat inactivation at 85 °C for 10 min. Then, 10 μl of barcoded reverse transcription reaction were pooled into a single tube; 1 µl per 50 µl pool volume of Thermolabile Exonuclease I (New England Biolabs) was then added and incubated for 4 min at 37 °C and 1 min at 80 °C. The cDNA from the pool was purified using in-house AMPure XP-like beads at a 1:1.4 sample-to-bead ratio and eluted in 50 µl nuclease-free water. Then 10 µl each of 1 M NaOH and 0.5 M EDTA, pH 8, were added to the purified sample and heated at 65 °C for 15 min to hydrolyze RNA in the RNA–cDNA hybrids. The reaction was purified using a Zymo Research Oligo Clean & Concentrator kit according to the manufacturer’s instructions for cDNA cleanup and eluted in 17 µl. A 23-µl reaction containing purified first-strand cDNA, 2.5 µl of Buffer 2 (New England Biolabs), 1 µl of 100 µM adapter-random hexamer primer and 200 nM deoxynucleoside triphosphate mix was heated to 95 °C for 1 min, followed by slow cooling to room temperature (0.1 °C s^−1^) in a PCR thermocycler. Second-strand synthesis was performed by adding 2 µl Klenow-Fragment DNA Polymerase (New England Biolabs) and incubating for 15 min at 30 °C. The reaction products were purified with a Zymo Research DNA Clean & Concentrator-5 Kit and eluted in 20 µl nuclease-free water. A final limited cycling PCR was performed using dual-indexing Illumina Primers (5 µM for each primer) and the Q5 NEBNext Ultra II Q5 Master Mix (New England Biolabs) for 10–14 cycles. The reaction was gel-purified, excising fragments between 200 and 900 bp and collected in 20 µl of elution buffer (10 mM TrisCl, pH 8). Libraries were sequenced on an Illumina NovaSeq S4 lane using a 150-bp pair-end mode.

### Pooled 3′-end bulk RNA sequencing analysis

For pooled 3′-end bulk RNA-seq, FASTQ read files were processed using the BRB-seqTools^57^ pipeline (https://github.com/DeplanckeLab/BRB-seqTools). First, read 1 was trimmed to 25 nucleotides using Cutadapt. Read 2 was then aligned to either the *R. aegyptiacus* reference genome (assembly mRouAeg1.p) or the human reference genome (assembly GRCh38.90) using STAR with the following parameters: --runMode alignReads,--genomeDir /path/to/STAR_index,--outFilterMultimapNmax 1,--outSAMtype BAM Unsorted,--outFileNamePrefix /path/to/output_folder, and --readFilesIn /path/to/read2.fastq. UMI-based gene count matrices were generated using the BRBSeq-CreateDGEMatrix tool, which combines the aligned read 2 BAM file with the trimmed read 1 FASTQ file containing the UMI and sample barcode information. The command included input files for trimmed read 1, BAM alignment, the sample barcode sheet and the gene annotation file (GTF), with parameters specifying UMI length and the output directory. The resulting gene expression matrix was used for downstream differential gene expression analysis in R using the edgeR package^58^. Genes with low expression were excluded from the analysis, specifically those with less than one CPM in at least two of three replicates in a given sample group (for example, mock-treated, IFN-treated or virus-infected). Trimmed mean of *M*-values normalization (TMM) was applied to raw counts. A gene-wise negative binomial generalized linear model was then fitted to the data using the glmQLFit function, incorporating sample group information. DEGs were identified using glmQLFTest and filtered for significance based on an absolute log fold change greater than 0.25 and a *P* < 0.05. DEG tables were used for visualization and statistical analysis in Prism (v.9). GO enrichment analysis of upregulated genes was performed using the clusterProfiler^59^ package in R. Exclusive and overlapping sets of DEGs were visualized with eulerr (https://eulerr.co/). TMM-normalized CPM were calculated using the edgeR cpm function with TMM-normalized counts as input.

The bulk and scRNA-seq data analysis is fully described in the Methods. Interested researchers are encouraged to contact the corresponding author(s) for additional details.

### Reanalyses of in vivo MARV Egyptian fruit bat infection data

Gene expression data from MARV-infected Egyptian fruit bats were reanalyzed by downloading the published nCounter-normalized dataset from ref. ^7^. A subset of the data was generated, focusing on the expression of ISGs and MARV genes in selected tissue samples, including the skin at the inoculation site, liver and colon.

### Statistical analysis

The statistical tests used are described in the corresponding figure legends. Data collection and analysis were not performed blind to the experimental conditions. Data distribution was assumed to be normal, but this assumption was not formally tested.

### Reporting summary

Further information on research design is available in the Nature Portfolio Reporting Summary linked to this article.

## Online content

Any methods, additional references, Nature Portfolio reporting summaries, source data, extended data, supplementary information, acknowledgements, peer review information; details of author contributions and competing interests; and statements of data and code availability are available at 10.1038/s41590-025-02155-1.

## Supplementary information

Reporting Summary

Supplementary Table 1 scRNA-seq cluster information for *R. aegyptiacus* lung and tracheal samples

Supplementary Table 2 scRNA-seq cluster information for *R. aegyptiacus* nasal and bronchial ALI cultures or bat alveolar organoids.

Supplementary Table 3 scRNA-seq cluster information for human nasal and bronchial ALI cultures.

Supplementary Table 4 scRNA-seq cluster information for *R. aegyptiacus* small intestinal tissue and small intestinal organoids.

Supplementary Table 5 Differential gene expression analysis for bat and human organoids stimulated with type I or III interferon.

Supplementary Table 6 Differential gene expression analysis for bat and human organoids infected with virus

Supplementary Table 7 Primer sequences used in this study.

## Data Availability

Raw and processed sequencing data are available at the Gene Expression Omnibus (GEO) under accession GSE291815. The scRNA-seq samples, including the Seurat objects presented in the study, can be accessed under the following accession numbers: bat and human SI organoids (GSM8842800); bat and human bronchial ALI cultures (GSM8842801); bat alveolar organoids (GSM8842802); bat and human nasal ALI cultures (GSM8842803); bat SI tissue (GSM8842804, GSM8842805, GSM8842806); bat lung tissue (GSM8842807, GSM8842808, GSM8842809, GSM8842810); bat trachea tissue (GSM8842811); pooled 3′-end bulk RNA-seq data, including demultiplexed raw UMI count matrices for bat SI organoids (GSM8842812); bat alveolar organoids (GSM8842813); bat nasal and intestinal organoids infected with MARV (GSM8842814); bat nasal organoids treated with IFN (GSM8842815); human SI organoids (GSM8842816); and human bronchial ALI cultures (GSM8842817). Reagents are available upon reasonable request and after signing a materials transfer agreement.

## References

[1] Irving, A. T., Ahn, M., Goh, G., Anderson, D. E. & Wang, L.-F. Lessons from the host defences of bats, a unique viral reservoir. *Nature* **589**, 363–370 (2021). 33473223 10.1038/s41586-020-03128-0

[2] Jebb, D. et al. Six reference-quality genomes reveal evolution of bat adaptations. *Nature* **583**, 578–584 (2020). 32699395 10.1038/s41586-020-2486-3 PMC8075899

[3] Zhang, G. et al. Comparative analysis of bat genomes provides insight into the evolution of flight and immunity. *Science* **339**, 456–460 (2013). 23258410 10.1126/science.1230835 PMC8782153

[4] Pavlovich, S. S. et al. The Egyptian rousette genome reveals unexpected features of bat antiviral immunity. *Cell* **173**, 1098–1110 (2018). 29706541 10.1016/j.cell.2018.03.070 PMC7112298

[5] Morales, A. E. et al. Bat genomes illuminate adaptations to viral tolerance and disease resistance. *Nature* **638**, 449–458 (2025). 39880942 10.1038/s41586-024-08471-0 PMC11821529

[6] Wang, L.-F., Gamage, A. M., Chan, W. O. Y., Hiller, M. & Teeling, E. C. Decoding bat immunity: the need for a coordinated research approach. *Nat. Rev. Immunol.* **21**, 269–271 (2021). 33649605 10.1038/s41577-021-00523-0 PMC7919622

[7] Guito, J. C. et al. Asymptomatic infection of Marburg virus reservoir bats is explained by a strategy of immunoprotective disease tolerance. *Curr. Biol.* **31**, 257–270 (2021). 33157026 10.1016/j.cub.2020.10.015

[8] Forero, A. et al. Differential activation of the transcription factor IRF1 underlies the distinct immune responses elicited by type I and Type III interferons. *Immunity* **51**, 451–464 (2019). 31471108 10.1016/j.immuni.2019.07.007 PMC7447158

[9] Gamage, A. M. et al. Single-cell transcriptome analysis of the in vivo response to viral infection in the cave nectar bat *Eonycteris spelaea*. *Immunity* **55**, 2187–2205 (2022). 36351376 10.1016/j.immuni.2022.10.008

[10] Whitsett, J. A. & Alenghat, T. Respiratory epithelial cells orchestrate pulmonary innate immunity. *Nat. Immunol.* **16**, 27–35 (2015). 25521682 10.1038/ni.3045 PMC4318521

[11] Schuh, A. J. et al. Natural reservoir *Rousettus aegyptiacus* bat host model of orthonairovirus infection identifies potential zoonotic spillover mechanisms. *Sci. Rep.* **12**, 20936 (2022). 36463252 10.1038/s41598-022-24673-w PMC9719536

[12] Halwe, N. J. et al. Egyptian fruit bats (*Rousettus aegyptiacus*) were resistant to experimental inoculation with avian-origin influenza A virus of subtype H9N2, but are susceptible to experimental infection with bat-borne H9N2 virus. *Viruses* **13**, 672 (2021). 33919890 10.3390/v13040672 PMC8070959

[13] Amman, B. R. et al. Experimental infection of Egyptian rousette bats (*Rousettus aegyptiacus*) with Sosuga virus demonstrates potential transmission routes for a bat-borne human pathogenic paramyxovirus. *PLoS Negl. Trop. Dis.* **14**, e0008092 (2020). 32119657 10.1371/journal.pntd.0008092 PMC7067492

[14] Hewitt, R. J. & Lloyd, C. M. Regulation of immune responses by the airway epithelial cell landscape. *Nat. Rev. Immunol.* **21**, 347–362 (2021). 33442032 10.1038/s41577-020-00477-9 PMC7804588

[15] Sachs, N. et al. Long-term expanding human airway organoids for disease modeling. *EMBO J.* **38**, e100300 (2019). 30643021 10.15252/embj.2018100300 PMC6376275

[16] Konishi, S., Tata, A. & Tata, P. R. Defined conditions for long-term expansion of murine and human alveolar epithelial stem cells in three-dimensional cultures. *STAR Protoc.* **3**, 101447 (2022). 35712012 10.1016/j.xpro.2022.101447 PMC9192963

[17] Van der Velden, J. L., Bertoncello, I. & McQualter, J. L. LysoTracker is a marker of differentiated alveolar type II cells. *Respir. Res.* **14**, 123 (2013). 24215602 10.1186/1465-9921-14-123 PMC3840660

[18] Becker, M. B., Zülch, A., Bosse, A. & Gruss, P. Irx1 and Irx2 expression in early lung development. *Mech. Dev.* **106**, 155–158 (2001). 11472847 10.1016/s0925-4773(01)00412-9

[19] Oliver, G. et al. *Six3*, a murine homologue of the *sine oculis* gene, demarcates the most anterior border of the developing neural plate and is expressed during eye development. *Development* **121**, 4045–4055 (1995). 8575305 10.1242/dev.121.12.4045

[20] Ualiyeva, S. et al. Tuft cell-produced cysteinyl leukotrienes and IL-25 synergistically initiate lung type 2 inflammation. *Science* **6**, eabj0474 (2021). 10.1126/sciimmunol.abj0474 PMC8750919 34932383

[21] Kazer, S. W. et al. Primary nasal influenza infection rewires tissue-scale memory response dynamics. *Immunity* **57**, 1955–1974 (2024). 38964332 10.1016/j.immuni.2024.06.005 PMC11324402

[22] Deprez, M. et al. A single-cell atlas of the human healthy airways. *Am. J. Respir. Crit. Care Med.* **202**, 1636–1645 (2020). 32726565 10.1164/rccm.201911-2199OC

[23] Kanoh, S., Tanabe, T. & Rubin, B. K. IL-13-induced MUC5AC production and goblet cell differentiation is steroid resistant in human airway cells. *Clin. Exp. Allergy* **41**, 1747–1756 (2011). 22092504 10.1111/j.1365-2222.2011.03852.x

[24] Chan, L. L. Y. et al. Generation of self-replicating airway organoids from the cave nectar bat *Eonycteris spelaea* as a model system for studying host–pathogen interactions in the bat airway epithelium. *Emerg. Microbes Infect.* **12**, e2148561 (2023). 36440480 10.1080/22221751.2022.2148561 PMC9754017

[25] Mabbott, N. A., Donaldson, D. S., Ohno, H., Williams, I. R. & Mahajan, A. Microfold (M) cells: important immunosurveillance posts in the intestinal epithelium. *Mucosal Immunol.* **6**, 666–677 (2013). 23695511 10.1038/mi.2013.30 PMC3686595

[26] Surve, M. V. et al. Single-cell transcriptomes, lineage, and differentiation of functional airway microfold cells. *Am. J. Respir. Cell Mol. Biol.* **69**, 698–701 (2023). 38038398 10.1165/rcmb.2023-0292LE PMC10704116

[27] Fujii, M. et al. Human intestinal organoids maintain self-renewal capacity and cellular diversity in niche-inspired culture condition. *Cell Stem Cell* **23**, 787–793 (2018). 30526881 10.1016/j.stem.2018.11.016

[28] Elmentaite, R. et al. Cells of the human intestinal tract mapped across space and time. *Nature* **597**, 250–255 (2021). 34497389 10.1038/s41586-021-03852-1 PMC8426186

[29] Beumer, J. et al. High-resolution mRNA and secretome atlas of human enteroendocrine cells. *Cell* **181**, 1291–1306 (2020). 32407674 10.1016/j.cell.2020.04.036

[30] Burclaff, J. et al. A proximal-to-distal survey of healthy adult human small intestine and colon epithelium by single-cell transcriptomics. *Cell. Mol. Gastroenterol. Hepatol.* **13**, 1554–1589 (2022). 35176508 10.1016/j.jcmgh.2022.02.007 PMC9043569

[31] Fung, K. Y. et al. Interferon-ε protects the female reproductive tract from viral and bacterial infection. *Science* **339**, 1088–1092 (2013). 23449591 10.1126/science.1233321 PMC3617553

[32] Jayaprakash, A. D. et al. Marburg and Ebola virus infections elicit a complex, muted inflammatory state in bats. *Viruses* **15**, 350 (2023). 36851566 10.3390/v15020350 PMC9958679

[33] Shaw, A. E. et al. Fundamental properties of the mammalian innate immune system revealed by multispecies comparison of type I interferon responses. *PLoS Biol.* **15**, e2004086 (2017). 29253856 10.1371/journal.pbio.2004086 PMC5747502

[34] Cuomo-Dannenburg, G. et al. Marburg virus disease outbreaks, mathematical models, and disease parameters: a systematic review. *Lancet Infect. Dis.* **24**, e307–e317 (2024). 38040006 10.1016/S1473-3099(23)00515-7 PMC7615873

[35] Schuh, A. J. et al. Modelling filovirus maintenance in nature by experimental transmission of Marburg virus between Egyptian rousette bats. *Nat. Commun.* **8**, 14446 (2017). 28194016 10.1038/ncomms14446 PMC5316840

[36] Lazear, H. M., Schoggins, J. W. & Diamond, M. S. Shared and distinct functions of type I and type III interferons. *Immunity* **50**, 907–923 (2019). 30995506 10.1016/j.immuni.2019.03.025 PMC6839410

[37] Friedrichs, V., Balkema-Buschmann, A., Dorhoi, A. & Pei, G. Selection and stability validation of reference gene candidates for transcriptional analysis in *Rousettus aegyptiacus*. *Sci. Rep.* **11**, 21662 (2021). 34737406 10.1038/s41598-021-01260-z PMC8568961

[38] Frolova, E. I. et al. Roles of nonstructural protein nsP2 and Alpha/Beta interferons in determining the outcome of Sindbis virus infection. *J. Virol.* **76**, 11254–11264 (2002). 12388685 10.1128/JVI.76.22.11254-11264.2002 PMC136776

[39] Guito, J. C. et al. Coordinated inflammatory responses dictate Marburg virus control by reservoir bats. *Nat. Commun.* **15**, 1826 (2024). 38418477 10.1038/s41467-024-46226-7 PMC10902335

[40] Lin, K. L. et al. Temporal characterization of Marburg virus Angola infection following aerosol challenge in rhesus macaques. *J. Virol.* **89**, 9875–9885 (2015). 26202230 10.1128/JVI.01147-15 PMC4577920

[41] Messaoudi, I., Amarasinghe, G. K. & Basler, C. F. Filovirus pathogenesis and immune evasion: insights from Ebola virus and Marburg virus. *Nat. Rev. Microbiol.* **13**, 663–676 (2015). 26439085 10.1038/nrmicro3524 PMC5201123

[42] Valmas, C. et al. Marburg virus evades interferon responses by a mechanism distinct from Ebola virus. *PLoS Pathog.* **6**, e1000721 (2010). 20084112 10.1371/journal.ppat.1000721 PMC2799553

[43] Edwards, M. R. et al. Differential regulation of interferon responses by Ebola and Marburg virus VP35 proteins. *Cell Rep.* **14**, 1632–1640 (2016). 26876165 10.1016/j.celrep.2016.01.049 PMC4767585

[44] Irving, A. T. et al. Interferon regulatory factors IRF1 and IRF7 directly regulate gene expression in bats in response to viral infection. *Cell Rep.* **33**, 108345 (2020). 33147460 10.1016/j.celrep.2020.108345 PMC8755441

[45] He, G.-W. et al. Optimized human intestinal organoid model reveals interleukin-22-dependency of Paneth cell formation. *Cell Stem Cell* **29**, 1333–1345 (2022). 36002022 10.1016/j.stem.2022.08.002 PMC9438971

[46] Arnold, C. E. et al. Transcriptomics reveal antiviral gene induction in the Egyptian rousette bat is antagonized in vitro by Marburg virus infection. *Viruses* **10**, 607 (2018). 30400182 10.3390/v10110607 PMC6266330

[47] Kraiczy, J. et al. DNA methylation defines regional identity of human intestinal epithelial organoids and undergoes dynamic changes during development. *Gut* **68**, 49–61 (2019). 29141958 10.1136/gutjnl-2017-314817 PMC6839835

[48] Güler-Gane, G. et al. Overcoming the refractory expression of secreted recombinant proteins in mammalian cells through modification of the signal peptide and adjacent amino acids. *PLoS ONE* **11**, e0155340 (2016). 27195765 10.1371/journal.pone.0155340 PMC4873207

[49] Fleming, S. J. et al. Unsupervised removal of systematic background noise from droplet-based single-cell experiments using CellBender. *Nat. Methods* **20**, 1323–1335 (2023). 37550580 10.1038/s41592-023-01943-7

[50] Wolock, S. L., Lopez, R. & Klein, A. M. Scrublet: computational identification of cell doublets in single-cell transcriptomic data. *Cell Syst.* **8**, 281–291 (2019). 30954476 10.1016/j.cels.2018.11.005 PMC6625319

[51] Stuart, T. et al. Comprehensive integration of single-cell data. *Cell* **177**, 1888–1902 (2019). 31178118 10.1016/j.cell.2019.05.031 PMC6687398

[52] Butler, A., Hoffman, P., Smibert, P., Papalexi, E. & Satija, R. Integrating single-cell transcriptomic data across different conditions, technologies, and species. *Nat. Biotechnol.* **36**, 411–420 (2018). 29608179 10.1038/nbt.4096 PMC6700744

[53] Levinger, R. et al. Single-cell and spatial transcriptomics illuminate bat immunity and barrier tissue evolution. *Mol. Biol. Evol.* **42**, msaf017 (2025). 39836373 10.1093/molbev/msaf017 PMC11817796

[54] Sikkema, L. et al. An integrated cell atlas of the lung in health and disease. *Nat. Med.* **29**, 1563–1577 (2023). 37291214 10.1038/s41591-023-02327-2 PMC10287567

[55] Heaton, H. et al. Souporcell: robust clustering of single-cell RNA-seq data by genotype without reference genotypes. *Nat. Methods* **17**, 615–620 (2020). 32366989 10.1038/s41592-020-0820-1 PMC7617080

[56] Bush, E. C. et al. PLATE-Seq for genome-wide regulatory network analysis of high-throughput screens. *Nat. Commun.* **8**, 105 (2017). 28740083 10.1038/s41467-017-00136-z PMC5524642

[57] Alpern, D. et al. BRB-seq: ultra-affordable high-throughput transcriptomics enabled by bulk RNA barcoding and sequencing. *Genome Biol.* **20**, 71 (2019). 30999927 10.1186/s13059-019-1671-x PMC6474054

[58] Robinson, M. D., McCarthy, D. J. & Smyth, G. K. edgeR: a Bioconductor package for differential expression analysis of digital gene expression data. *Bioinformatics* **26**, 139–140 (2010). 19910308 10.1093/bioinformatics/btp616 PMC2796818

[59] Yu, G., Wang, L.-G., Han, Y. & He, Q.-Y. clusterProfiler: an R package for comparing biological themes among gene clusters. *OMICS* **16**, 284–287 (2012). 22455463 10.1089/omi.2011.0118 PMC3339379

