# Supplementary material for: Bat organoids reveal antiviral responses at epithelial surfaces
Source: Nat Immunol. 2025 May 21;26(6):934–46. doi: 10.1038/s41590-025-02155-1 (PMC12133567; doi:10.1038/s41590-025-02155-1)
Supplement: Supplementary file 1 — Reporting Summary [file 41590_2025_2155_MOESM1_ESM.pdf]

nature portfolio

Corresponding author(s): Josef M. Penninger and Max J. Kellner

Last updated by author(s): Apr 5, 2025

Reporting Summary

Nature Portfolio wishes to improve the reproducibility of the work that we publish. This form provides structure for consistency and transparency in reporting. For further information on Nature Portfolio policies, see our Editorial Policies and the Editorial Policy Checklist.

Statistics

For all statistical analyses, confirm that the following items are present in the figure legend, table legend, main text, or Methods section.

n/a	Confirmed	
☐	☒	The exact sample size (n) for each experimental group/condition, given as a discrete number and unit of measurement
☐	☒	A statement on whether measurements were taken from distinct samples or whether the same sample was measured repeatedly
☐	☒	The statistical test(s) used AND whether they are one- or two-sided<br>*Only common tests should be described solely by name; describe more complex techniques in the Methods section.*
☒	☐	A description of all covariates tested
☒	☐	A description of any assumptions or corrections, such as tests of normality and adjustment for multiple comparisons
☐	☒	A full description of the statistical parameters including central tendency (e.g. means) or other basic estimates (e.g. regression coefficient) AND variation (e.g. standard deviation) or associated estimates of uncertainty (e.g. confidence intervals)
☐	☒	For null hypothesis testing, the test statistic (e.g. F, t, r) with confidence intervals, effect sizes, degrees of freedom and P value noted<br>*Give P values as exact values whenever suitable.*
☒	☐	For Bayesian analysis, information on the choice of priors and Markov chain Monte Carlo settings
☒	☐	For hierarchical and complex designs, identification of the appropriate level for tests and full reporting of outcomes
☒	☐	Estimates of effect sizes (e.g. Cohen's d, Pearson's r), indicating how they were calculated

Our web collection on statistics for biologists contains articles on many of the points above.

Software and code

Policy information about availability of computer code

Data collection

Cellranger (version 7.1), cellbender (version 0.3.0), souporcell (version 2.0), BRBseqTools (version 1.5), GraphPad Prism (version 9), R (R version 4.4.1), R packages: Seuratv4 (version 4.2.1), edgeR (version 4.2.1), (dplyr 1.1.4), clusterProfiler (version 4.12.6).

Data analysis

For Rousettus aegyptiacus lung trachea and small intestine libraries, sequencing reads from 10x Genomics libraries were processed using Cell Ranger (v7.1). A custom genome index was created using cellranger mkref with the Rousettus aegyptiacus reference genome (Assembly mRouAeg1.p). Gene expression count matrices were generated using cellranger count. Next, filtered and denoised count matrices were produced using CellBender (cellbender remove-background), with the following parameters: --expected-cells 20000 --total-droplets-included 30000 --fpr 0.01 --epochs 150. Doublets were identified using Scrublet on the filtered gene expression matrix (expected doublet rate = 0.1). Downstream analysis was conducted in R using Seurat (v4.2.1). Gene expression matrices in .h5 format were imported using Read10X_h5 to generate individual Seurat objects. Doublets identified by Scrublet were removed. Seurat objects were filtered to retain cells with >300 and <40,000 detected genes per cell, and data were log-normalized (NormalizeData). Cell cycle scores (G2M and S phase via CellCycleScoring) were computed and regressed out during scaling (ScaleData). The top 2000 variable features per sample were identified (FindVariableFeatures), and integration anchors were calculated using FindIntegrationAnchors. Samples were integrated with Seurat CCA (IntegrateData), followed by data scaling and PCA (RunPCA). The number of PCs for UMAP dimensionality reduction was selected via ElbowPlot. Cell clusters were identified using FindNeighbours and FindClusters (resolutions 0.5–1). Marker genes were identified with FindAllMarkers (parameters: min.pct = 0.25, log.fc = 0.25, only.pos = TRUE). Clusters were annotated based on known marker genes (see refs 28,53,54) (Clustering results in Supplementary Tables 1, 4). A subset containing epithelial cell types was extracted to obtain epithelial cells only. For bat lung and trachea, the clustering/annotation pipeline was repeated to generate a reference dataset. The small intestine epithelial dataset (SIT) was further integrated together with bat SIORG (see below).

For organoid libraries (bat and human NasalALI, bat and human BronchialALI, bat AlvORG, and bat and human SIORG), sequencing reads were

processed using Cell Ranger (v7.1) with a custom reference index built from the Rousettus aegyptiacus (mRouAeg1.p) and Homo sapiens (GRCh38.90) genomes, generated using cellranger mkref. For bat AlvORG samples, a custom index containing only the R. aegyptiacus (mRouAeg1.p) genome was used. Cellbender remove-background was applied with the same parameters as described above for tissue samples. Souporcell (souporcell_pipeline.py) was used to cluster cells by SNPs using aligned BAM files (possorted_genome_bam.bam) with parameters: -k X, -t 8, --skip_remap True, --ignore True. For downstream analysis, individual Seurat objects were created per 10x library (e.g., human + bat SIORG, human + bat NasalALI, human + bat BronchialALI, bat AlvORG). Metadata from Cell Ranger and Souporcell were added; multiplets and unassigned cells were excluded. Individual donor samples (n = 3 for bats) were isolated using Souporcell metadata information (SplitObject), and three integration sets were analyzed: Bat NasalALI+ BronchialALI (3 donors each, 6 samples), bat AlvORG (3 donors), and Bat SIORG (2 donors) + SIORG-DIFF + SIT (4 samples). Each dataset was processed as follows: Cells with >500 detected genes were retained, followed by log-normalization. Cell cycle scores were regressed out during data scaling. Top 2000 variable genes were selected. Integration anchors were identified using FindIntegrationAnchors, and datasets were integrated with IntegrateData. PCA was performed, and UMAP dimensions were chosen based on ElbowPlot (typically PCs 1–30). Clusters were identified (FindNeighbours, FindClusters), and marker genes computed (FindAllMarkers). Cell cluster annotation was performed manually using well-established marker genes for the mammalian airway and intestinal epithelium (see refs 28,53,54) (Clustering results in Supplementary Table 2).

To refine rare cell-types in bat airway organoids, $GP2^{+}$ microfold cells were identified within the ciliated/microfold cluster based on non-zero expression of GP2. Ionocytes were annotated based on the expression of FOXI1, ASCL3 or PDE1C (only in NasalALI; BronchialALI counterparts lacked expression and were therefore reassigned as suprabasal/secretory cells). Neuroendocrine cells expressing CHGA, CHGB or SCG3 were detected in NasalALI only, while cells from BronchialALI in that cluster that did not were similarly reassigned to the suprabasal/secretory lineage. In SIORG and SIT datasets, enteroendocrine cells (EECs) were identified by CHGA and CHGB expression were subset and reclustered following the Seurat's guided clustering vignette.

Human datasets (NasalALI, BronchialALI, and SIORG) were analysed separately following Seurat's online guided clustering vignette, and clusters were annotated based on established marker genes (see refs 27,28,54) (Clustering results in Supplementary Table 3).

For comparing gene expression across different bat organoid models, Seurat objects were merged into a single object following count normalization using SCTransform. For cross-species comparisons between human and bat organoids, a list of 1:1 orthologs was generated, and merged Seurat objects were filtered to retain orthologs only. This involved extracting raw counts using GetAssayData, filtering for orthologs, and reconstructing the Seurat object with the filtered count matrix and associated metadata. Expression data were then normalized using SCTransform, and cell identities were assigned based on species. Differentially expressed genes were identified using FindMarkers (ident.1 = bat, ident.2 = human, min.pct = 0.25, log.fc = 0.25). Plots were generated in GraphPad Prism (v9). Interferon-stimulated gene (ISG) scores were calculated using Seurat's AddModuleScore function, with a published list of conserved mammalian ISGs (obtained from ref 33) as input.

For pooled 3′-end bulk RNA-seq, FASTQ read files were processed using the BRB-seqTools pipeline (https://github.com/DeplanckeLab/BRB-seqTools). First, Read 1 was trimmed to 25 nucleotides using Cutadapt. Read 2 was then aligned to either the Rousettus aegyptiacus reference genome (Assembly mRouAeg1.p) or the human reference genome (Assembly GRCh38.90) using STAR with the following parameters: --runMode alignReads, --genomeDir /path/to/STAR_index, --outFilterMultimapNmax 1, --outSAMtype BAM Unsorted, --outFileNamePrefix /path/to/output_folder, and --readFilesIn /path/to/read2.fastq. UMI-based gene count matrices were generated using the BRBSeq-CreateDGEMatrix tool, which combines the aligned Read 2 BAM file with the trimmed Read 1 FASTQ file containing UMI and sample barcode information. The command included input files for trimmed Read 1, BAM alignment, sample barcode sheet, and the gene annotation file (GTF), with parameters specifying the UMI length and output directory. The resulting gene expression matrix was used for downstream differential gene expression analysis in R using the edgeR package. Genes with low expression were excluded from the analysis—specifically, those with fewer than 1 count per million (CPM) in at least two out of three replicates within a given sample group (e.g., mock-treated, interferon-treated, or virus-infected). TMM normalization was applied to raw counts. A genewise negative binomial generalized linear model was then fitted to the data using the glmQLFit function, incorporating sample group information. Differentially expressed genes (DEGs) were identified using glmQLFTest and filtered for significance based on an absolute log fold change greater than 0.25 and a p-value less than 0.05. DEG tables were used for visualization and statistical analysis in GraphPad Prism (version 9). Gene ontology enrichment analysis of upregulated genes was performed using the clusterProfiler package in R. Exclusive and overlapping sets of DEGs were visualized eulerr (https://eulerr.co). Normalized counts per million (CPM) were calculated using the edgeR cpm function with TMM-normalized counts as input.

For manuscripts utilizing custom algorithms or software that are central to the research but not yet described in published literature, software must be made available to editors and reviewers. We strongly encourage code deposition in a community repository (e.g. GitHub). See the Nature Portfolio guidelines for submitting code & software for further information.

Data

Policy information about availability of data

All manuscripts must include a data availability statement. This statement should provide the following information, where applicable:

- Accession codes, unique identifiers, or web links for publicly available datasets
- A description of any restrictions on data availability
- For clinical datasets or third party data, please ensure that the statement adheres to our policy

Raw and processed sequencing data are available under NCBI GEO experiment (accession GSE291815). All steps necessary to reproduce processing of raw data are clearly describes in the results.

Research involving human participants, their data, or biological material

Policy information about studies with human participants or human data. See also policy information about sex, gender (identity/presentation), and sexual orientation and race, ethnicity and racism.

Reporting on sex and gender: Donors were either male or female and age ranged from 20-50 years.

Reporting on race, ethnicity, or other socially relevant groupings	Ethnicity was no study criteria and not recorded, but expected to be primary Caucasian.
Population characteristics	This study focuses on bat organoids and their unique antiviral immune responses. Human airway epithelial cells (commercial for bronchial and nasal brush biopsy) were included solely as a reference for comparative purposes. These cells were derived from healthy adult donors within the age range of 20–50 years. Given the use of only one individual and the limited scope of human data, covariate-relevant population characteristics are not applicable to the study's design or findings, which center on bat-derived epithelial organoid models.
Recruitment	For duodenum organoids obtained from Zilbauer lab: Intestinal biopsies were collected from human patients undergoing routine endoscopy following ethical approval (REC-12/EE/0482) and informed consent.<br>For nasal epithelial cells: Human nasal epithelial cells were collected from a brush biopsy of the mid-turbinate section following ethical approval (Votum ECS 2234/2021) and informed consent.
Ethics oversight	Experiments using human nasal epithelial cells were approved by the ethic commission of the Medical University of Vienna (Votum ECS 2234/2021). Human small intestinal organoids were established from healthy duodenum biopsy specimens by isolating intestinal crypts (full ethical approval from the NRES Committee East of England, Hertfordshire (REC-12/EE/0482))

Note that full information on the approval of the study protocol must also be provided in the manuscript.

Field-specific reporting

Please select the one below that is the best fit for your research. If you are not sure, read the appropriate sections before making your selection.

☒ Life sciences ☐ Behavioural & social sciences ☐ Ecological, evolutionary & environmental sciences

For a reference copy of the document with all sections, see nature.com/documents/nr-reporting-summary-flat.pdf

Life sciences study design

All studies must disclose on these points even when the disclosure is negative.

Sample size	For bat organoid experiments, we were limited in samples number and thus used the maximum available sample size for our experiments (three donors for full characterization).
Data exclusions	No data was excluded
Replication	Experiments involved in the study were performed in at least three biological replicates, that is individual donor samples. Alternatively, replicates refer to different organoid batches or technical replicate measurements. The type of replicate is mentioned at the respective section of the manuscript. All technical and biological replications of experiments were successful.
Randomization	Randomization was not relevant for this study. Experimental groups were defined based on species or treatment (bat vs human, infected vs uninfected, treated vs non-treated)
Blinding	Blinding was not relevant for this study. Human judgment or subjective interpretation was not involved in any step of the experiment (data collection, observation, analysis) and the experimental design ensures minimal influence of cognitive bias

Reporting for specific materials, systems and methods

We require information from authors about some types of materials, experimental systems and methods used in many studies. Here, indicate whether each material, system or method listed is relevant to your study. If you are not sure if a list item applies to your research, read the appropriate section before selecting a response.

Materials & experimental systems

n/a	Involved in the study	
☐	☒	Antibodies
☐	☒	Eukaryotic cell lines
☒	☐	Palaeontology and archaeology
☐	☒	Animals and other organisms
☒	☐	Clinical data
☒	☐	Dual use research of concern
☒	☐	Plants

Methods

n/a	Involved in the study	
☒	☐	ChIP-seq
☒	☐	Flow cytometry
☒	☐	MRI-based neuroimaging

Antibodies

Antibodies used	The following antibodies were used: anti-KRT5 (Rabbit polyclonal antibody, Sigma-Aldrich, Cat. # SAB4501651, dilution 1:200), anti-acetylated α Tubulin (Mouse monoclonal IgG2b antibody, clone 6-11B-1, SCBT Cat. # sc-23950, dilution 1:500), anti-AVIL (Rabbit polyclonal antibody, Thermofisher cat. # PA5-90703, dilution 1:200), anti-SFTPC (Rabbit polyclonal antibody, Thermofisher cat. # PA5-71680, dilution 1:200), anti-E-Cadherin (Mouse monoclonal IgG2a antibody, Clone 36, BD Biosciences, Cat. # 610182), anti-IFN-epsilon (Monoclonal Mouse IgG2B antibody, Clone # 983338, RnD Systems Cat. # MAB9147-100, dilution 1:200). Secondary antibodies: Donkey anti-Rabbit IgG (H+L) Highly Cross-Adsorbed Secondary Antibody, Alexa Fluor™ 488, Thermofisher, Cat # A-21206, dilution 1:500), Donkey anti-Mouse IgG (H+L) Highly Cross-Adsorbed Secondary Antibody, Alexa Fluor™ 488, Thermofisher, Cat # A-21202, dilution 1:500), Donkey anti-Mouse IgG (H+L) Highly Cross-Adsorbed Secondary Antibody, Alexa Fluor™ 647, Thermofisher,Cat # A-31573, dilution 1:500), Donkey anti-Rabbit IgG (H+L) Highly Cross-Adsorbed Secondary Antibody, Alexa Fluor™ 647, Thermofisher, Cat # A-31571, dilution 1:500
Validation	All antibodies were of commercial source and valiated by the manufacture according to the data sheet. In-house we further validated antibodies: Primary antibodies were validated by 1) secondary antibody staining only, 2) Isotype control wherever appropriate, 3) use of sample with expected absent expression based on RNA-seq data (e.g., SFTPC in gut, IFNE in liver).

Eukaryotic cell lines

Policy information about cell lines and Sex and Gender in Research

Cell line source(s)	BHK-21 cells (ATCC CCL-10), VeroE6 (ATCC, CRL-1586). Novel epithelial organoids from captive-bred bats were derived from individual animals. 2D monolayer epithelial cell lines were further derived from the organoid lines. Human nasal epithelial cells were established from a nasal brush. Human bronchial epithelial cells were commercially obtained from Promocell (PC-C-12640, Lot 446Z036.9). Human small intestinal organoids were provided by the Zilbauer lab under an MTA.
Authentication	Organoids were sequenced at single level, showing species authenticity and cell identity
Mycoplasma contamination	Organoids and cells were routinely tested for absence of mycoplasma using an in-house PCR based test.
Commonly misidentified lines<br>(See ICLAC register)	not applicable

Animals and other research organisms

Policy information about studies involving animals; ARRIVE guidelines recommended for reporting animal research, and Sex and Gender in Research

Laboratory animals	Rousettus aegyptiacus bat tissue for organoid generation was derived from a breeding colony at the Friedrich-Loeffler-Institute in Germany (bat juvenile to adult). Routine organ harvest was done from juvenile or adult male or female bats in accordance with current European and national animal welfare regulations of the federal state of Mecklenburg-Western Pomerania, Germany
Wild animals	No wild animals were used in this study
Reporting on sex	Sex was not considered for the study but bat organoids were prepared from animals of both sexes.
Field-collected samples	No field collected samples were used.
Ethics oversight	No ethical approval was needed for routine organ harvest of captive-bred fruit bats.

Note that full information on the approval of the study protocol must also be provided in the manuscript.

Plants

Seed stocks	No plants were used in the study
Novel plant genotypes	Not applicable
Authentication	Not applicable
